# The Inactivation Principle: Mathematical Solutions Minimizing the Absolute Work and Biological Implications for the Planning of Arm Movements

**DOI:** 10.1371/journal.pcbi.1000194

**Published:** 2008-10-24

**Authors:** Bastien Berret, Christian Darlot, Frédéric Jean, Thierry Pozzo, Charalambos Papaxanthis, Jean Paul Gauthier

**Affiliations:** 1 Université de Bourgogne, INSERM U887 Motricité-Plasticité, Dijon, France; 2 Centre National de la Recherche Scientifique et École Nationale Supérieure des Télécommunications, CNRS/ENST, Paris, France; 3 École Nationale Supérieure de Techniques Avancées, ENSTA/UMA, Paris, France; 4 Istituto Italiano di Tecnologia, IIT, Genova, Italy; 5 IUT de Toulon, Université de Toulon, UMR 6168, La Garde, France; University College London, United Kingdom

## Abstract

An important question in the literature focusing on motor control is to determine which laws drive biological limb movements. This question has prompted numerous investigations analyzing arm movements in both humans and monkeys. Many theories assume that among all possible movements the one actually performed satisfies an optimality criterion. In the framework of optimal control theory, a first approach is to choose a cost function and test whether the proposed model fits with experimental data. A second approach (generally considered as the more difficult) is to infer the cost function from behavioral data. The cost proposed here includes a term called the absolute work of forces, reflecting the mechanical energy expenditure. Contrary to most investigations studying optimality principles of arm movements, this model has the particularity of using a cost function that is not smooth. First, a mathematical theory related to both direct and inverse optimal control approaches is presented. The first theoretical result is the Inactivation Principle, according to which minimizing a term similar to the absolute work implies simultaneous inactivation of agonistic and antagonistic muscles acting on a single joint, near the time of peak velocity. The second theoretical result is that, conversely, the presence of non-smoothness in the cost function is a necessary condition for the existence of such inactivation. Second, during an experimental study, participants were asked to perform fast vertical arm movements with one, two, and three degrees of freedom. Observed trajectories, velocity profiles, and final postures were accurately simulated by the model. In accordance, electromyographic signals showed brief simultaneous inactivation of opposing muscles during movements. Thus, assuming that human movements are optimal with respect to a certain integral cost, the minimization of an absolute-work-like cost is supported by experimental observations. Such types of optimality criteria may be applied to a large range of biological movements.

## Author Summary

When performing reaching and grasping movements, the brain has to choose one trajectory among an infinite set of possibilities. Nevertheless, because human and animal movements provide highly stereotyped features, motor strategies used by the brain were assumed to be optimal according to certain optimality criteria. In this study, we propose a theoretical model for motor planning of arm movements that minimizes a compromise between the absolute work exerted by the muscles and the integral of the squared acceleration. We demonstrate that under these assumptions agonistic and antagonistic muscles are inactivated during overlapping periods of time for quick enough movements. Moreover, it is shown that only this type of criterion can predict these inactivation periods. Finally, experimental evidence is in agreement with the predictions of the model. Indeed, we report the existence of simultaneous inactivation of opposing muscles during fast vertical arm movements. Therefore, this study suggests that biological movements partly optimize the energy expenditure, integrating both inertial and gravitational forces during the motor planning process.

## Introduction

In order to perform accurate goal-directed movements, the Central Nervous System (CNS) has to compute neural commands according to the initial state of the body, the location of the target, and the external forces acting on the limbs. Arm movement planning requires solving redundancy problems related to angular displacements, joint torques, muscular patterns, and neural inputs [1].

Experimental studies reported stereotypical kinematic features during pointing and reaching arm movements (e.g., quasi-straight finger paths, bell-shaped finger velocity profiles [2]–[4]). These features were found to be robust despite changes in mass, initial/final positions, amplitudes, and speeds of displacements [5]–[9].

Therefore, many studies have attempted to identify the principles of motion planning and control, hypothesizing that movements were optimal with respect to some criteria. The present article addresses the question whether motor planning is optimal according to an identifiable criterion.

A promising approach to answer this question, called *inverse* optimal control, is to record experimental data and try to infer a cost function with regard to which the observed behavior is optimal [10]. In the theory of linear-quadratic control, the question of which quadratic cost is minimized in order to control a linear system along certain trajectories was already raised by R. Kalman [11]. Some methods allowed deducing cost functions from optimal behavior in system and control theory (linear matrix inequalities, [12]) and in Markov decision processes (inverse reinforcement learning, [13]). In the field of sensorimotor control and learning, some authors suggested that motor learning results from the optimization of some “loss function” related to the task (e.g., pointing accuracy) providing, therefore, a technique allowing to measure such function from experimental data [14].

Nevertheless, in most optimal control studies focusing on arm movements, a cost function is chosen and used in a mathematical model to check its validity *a posteriori* by comparing the theoretical predictions to the experimental observations.

Kinematic models include minimum hand acceleration [15] and minimum hand jerk criteria [16]. These models produce horizontal arm movements that globally fit well with experimental data, providing smooth symmetric velocity profiles and straight trajectories in space. Dynamic models include minimum torque-change [17] and minimum commanded torque-change [18] criteria. They also accurately reproduce certain types of movements (point-to-point and via-point movements performed in the horizontal plane) but in several cases provide non-realistic double-peaked speed profiles (see for instance Figure 11 in [19]). In the Riemannian geometry framework, a model used geodesics to separately determine the geometrical and temporal movement features, allowing therefore a unification of previous computational models [19]. Specifically, the geodesic model accurately predicts the spatiotemporal features of three dimensional arm movements. However it results in hand paths that are excessively curved for planar movements. Additional criteria have also been considered, such as energy-like criteria [20]–[25] and effort related criteria [26], which minimize the peak value of the work, the metabolic energy expenditure, or the amount of neural control signals necessary to drive the arm. These models quantitatively reproduce some specific features of reaching and grasping, such as trajectories, velocity profiles, or final postures. Stochastic models, which are grounded on the hypothesis that noise in the nervous system corrupts command signals, have also been proposed. The minimum variance model was aimed at minimizing endpoint errors and provides not only accurate simulated trajectories of both eye saccades and arm pointing movements in the horizontal plane, but also the speed-accuracy trade-off described by Fitt's law [27]. In the optimal feedback control theory, noise is assumed to induce movement inaccuracy. If errors interfere with task goals, then the controller corrects deviations from the average trajectory. Otherwise the errors are ignored and, thus, variability in task-irrelevant dimensions is allowed [28]–[30].

Despite extensive literature concerning *direct* optimal control of arm movements, the hypotheses seem too restrictive in some models. For instance, in several models [19],[26], the static (gravity-related) and dynamic (speed-related) torques are calculated separately; therefore their predictions are independent from the gravity field. This assumption partly relies on the physiological observations that muscle activity patterns show two components: a tonic one (gravity-related) and a phasic one (speed-related) [31],[32]. Nevertheless, some authors reported difficulties in solving optimal control problems while taking into account gravitational forces in the optimization process [33],[34]. Thus, this assumption was also aimed at simplifying computations. Furthermore, the models previously cited are generally not consistent with the observation that the kinematics of arm movements performed in the sagittal plane depends on the direction with respect to gravity (i.e., upward versus downward movements) [35]–[38] whereas such a directional difference is significantly attenuated in microgravity [39].

A possible explanation of these findings would be that the CNS uses the gravity to move the limbs efficiently, rather than simply offset it at each instant. This idea guided the development of the theoretical model presented here. During a movement, the energetic consumption is related to the work of muscular forces. However, work is a signed physical quantity that may cancel itself out, even though both active and resistive forces consume energy in muscles. Therefore, work has to be always counted positive in order to express the energy expenditure of a movement: this is the *absolute work* of forces. The problem of minimizing this absolute work was never solved previously, despite its apparent simplicity and its potential interest for neurophysiologists. A reason might be the mathematical difficulty due to the non-differentiability of the cost function (induced by the absolute value function). Thus, while most existing models deal with smooth cost functions (i.e., functions that have continuous derivatives up to some desired order), this study relies on this non-smoothness property. The cost chosen here includes two terms: the first represents the absolute work and the second is proportional to the integral of the squared acceleration.

In this article, two theoretical results are reported. Firstly, an “Inactivation Principle” states that minimizing a cost similar to the absolute work implies the presence of simultaneous inactivation of both agonistic and antagonistic muscles acting on a joint during fast movements. Secondly, a reciprocal result is that the presence of such inactivation along optimal trajectories implies the non-smoothness of the cost function. Therefore, by using transversality arguments from Thom's Differential Topology [40], Pontryagin's Maximum Principle [41], and Non-smooth Analysis [42], an equivalence between the non-smoothness of the cost function and the presence of simultaneous inactivation of both agonistic and antagonistic muscles is established. The proposed model permits to simulate accurately the kinematics of fast vertical arm movements with one, two, and three degrees of freedom. Moreover, experimental observations actually show simultaneous silent periods on the electromyographic (EMG) signals of opposing muscles during fast arm movements.

## Results

The main results of this study are presented in the next two subsections. The theoretical analysis is exposed in the first subsection. In order to check the model, features of human arm movements were measured and are compared with the model predictions in the second subsection.

### Theoretical Analysis

The current subsection summarizes the mathematical theory which is more fully presented in the Materials and Methods Section. The reader who may not be interested in the full mathematical development of the model may read this subsection only, as a general survey.

#### Control systems

The mechanical systems of articulated segments considered here move in the gravity field and are controlled by external forces produced by muscles. In practice, vertical arm movements are considered with one, two, and three degrees of freedom (denoted by 1-dof, 2-dof, and 3-dof, respectively).

The equation describing a fully-actuated mechanical system (Σ) has the general form:
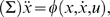
(1)where the control *u* (the forces or torques) acts on the acceleration vector of generalized coordinates 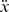, with at least as many control variables (*u_i_*)*_i_*_ = 1..*m*_ as the number *n* of degrees of freedom of the system. When considering agonistic-antagonistic pairs of muscles, it will happen that *m*>*n*, precisely *m* = 2*n*, i.e., one agonistic and one antagonistic muscle for each degree of freedom.

However, for the sake of simplicity, in the rest of the study, the assumption will be that *m* = *n* which means that the control variables consist of the net forces or torques acting on each joint.

Moreover, we assume that:

- *x* belongs to 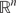 (or to a more general object: a *n*-dimensional differentiable manifold).
- *u* belongs to a subset *U* of 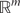 with 0 ∈ *intU* (the notation *intU* means the “interior” of the subset *U*).

Since there are physiological bounds on the forces produced by muscles, *U* is a product of intervals of the type:
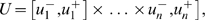
if the system is exactly-fully-actuated, or:
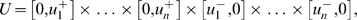
in the case of a pair of agonistic-antagonistic muscles for each degree of freedom. In both cases 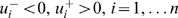.

- In the case *m* = *n*, *ϕ* is smooth, i.e., 
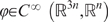
, and such that the Jacobian matrix 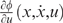 is always invertible.

Then, in order to get the general control systems, we set *X* = (*x*, *y*) = (*x*, *x* ˙) and rewrite the system as:
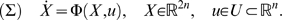
(2)


#### Optimal control problem

Here, pointing movements between two targets are defined by their duration *T* and by a pair of initial and final conditions (*x_s_*,*x_t_*) in the configuration space. The limb moves from *x_s_* to *x_t_*, starting and ending with zero velocity.

Movements are assumed to be optimal with respect to a certain integral cost of the form:
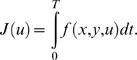
(3)


In the paper *f* is referred to as the *cost function*. The term *J* is called the *integral cost* or simply the *cost*. It is sometimes referred to as the *optimality criterion*.

The aim is to find the control *u* (e.g., the torques) and the corresponding admissible trajectory *X* that minimizes the above integral cost. An efficient way to solve this kind of problem is to use Pontryagin's Maximum Principle [41]. A statement of this principle is provided in the mathematical part of the Materials and Methods Section.

*Remark 1.* (1) A simplifying assumption is that the duration *T* of the motion is fixed. This is not essential, since: (i) Pontryagin's Maximum Principle also allows to deal with free movement durations: the time *T* is then determined by a supplementary condition of optimality, see [41]; (ii) as in [26], one could search for the time *T* that leads to a given amount of the integral cost. Here, the latter approach is better suited because the optimal cost will be a strictly decreasing function of *T* (see Theorem 1 in [43]). (2) Movements are driven in the configuration space, and positions of targets are defined in practice by their coordinates in the Cartesian space. There is a one-to-one relationship between target coordinates and limb configuration for 1-dof and 2-dof planar movements, but not for 3-dof planar movements. In this case, an infinity of final postures is compatible within the reach of a target in task-space. Nevertheless a solution can be found once again by mean of Pontryagin's Maximum Principle using *transversality conditions* [41]. (3) Since this study focuses on the command of transient movements, the questions of transition between posture and movement and stability of the final posture are not addressed. Nevertheless, it will happen that we consider the dynamics of muscles in the Mathematical Theory Subsection. In this case, the controls become motor orders sent by the motoneurons to each muscle. Thus, the initial and final torques necessary to maintain the arm at equilibrium are specified in this optimal control problem.

In order to study the control of movements by means of optimal control theory, various functions *f* were proposed previously in the literature. These functions, such as the famous minimum jerk [16] and minimum torque change [17], were generally smooth functions. Nevertheless in our case a non-smooth cost function appeared more suitable.

For actuated mechanical systems, the physical quantity that measures energy is the work of forces. However, the work of a force pulling in the direction arbitrarily defined as positive may cancel with the work of the force pulling in the opposite direction. Thus, the *absolute work* measures the energy expenditure of a movement. Indeed, the work of both the agonistic and antagonistic muscles requires a consumption of energy, provided by the hydrolysis of ATP to ADP, a physiological process taking place in muscle cells. The Mathematical Theory Subsection gives a precise definition of the absolute work *Aw*, which can be expressed as:
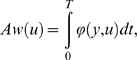
but the function *ϕ* is *not smooth*: it contains some “absolute values” that are Lipschitz-continuous, but non-differentiable at *u* = 0. For instance in the 1-dof case, *ϕ*(*y*,*u*) = |*yu*| where *y* is the angular velocity and *u* is the net torque. The absolute work term counts the mechanical energy actually spent to control the system (Σ).

Such a similar non-smooth cost function has been proposed by other authors [23] and thus it appears that the non-smoothness of the cost function arises naturally in motion planning problems. It is worthy to note that this is not an artificial mathematical construction.

*Remark 2.* An intuitive (but different) reason for considering non-smooth (or even discontinuous) cost functions in optimal control studies of arm movements could be that the forces acting on a joint result from distinct muscles.

In this study, the integral cost is assumed to have the general form:
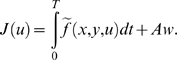
(4)


This expression represents a compromise between the absolute work *Aw* and some other *comfort term* defined by the function 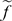. The terminology comfort term is purposely left vague. For instance (non-exhaustive list), one may choose the acceleration squared (as in [15]) or the torque squared (as in [23]) for the function 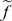.

This additional term is not crucial. One could assume that the CNS only minimizes the absolute work, but it seems to also minimize some integral costs accounting for the smoothness or precision of the movements [15]–[17],[27]. While the definition of the mechanical energy spent is well established, what should be the comfort term is more subjective. It may suggest that the motor system would avoid large accelerations, so as not to expose tendons and articulations to large jerks.

Here, in all examples and simulations, we will assume that 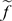 is proportional to the acceleration squared. For instance in the 1-dof case, 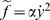 where *α* is a strictly positive constant. In that case, the term 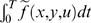 is just the *acceleration energy* in the sense of signal processing and will be denoted by *Ae*.

#### Theoretical results

An important concept in this study is that of inactivation.

*Definition 1.* A *partial inactivation* (or simply *inactivation*) is an occurrence during a certain strictly positive time-interval of an optimal trajectory corresponding to *u_i_* = 0 for some *i*, i.e., the *i*th control is zero during this time-interval. A *total inactivation* is a simultaneous inactivation of all controls.

Here, the controls (*u_i_*)*_i_*_ = 1..*n*_ are just the net torques applied at each joint.

An important theoretical result is what we call the *Inactivation Principle*. In mathematics, a principle is more than just a theorem. It is a statement of a general result that can be made true in different contexts, or more precisely transformed into a theorem under rather different types of technical assumptions.

***Inactivation Principle.*** *Minimizing a cost of type given by* *Equation 4* *implies the presence of stable partial inactivation in all nontrivial (nonequilibria) pointing movements for T sufficiently short (i.e., there is a time threshold for partial inactivation to occur). Moreover, there are stable optimal trajectories that contain total inactivation.*

This principle can be made very general and requires rather weak assumptions (see Remark 3 in the Mathematical Theory Subsection). The proof relies on arguments from non-smooth analysis [42], and is in the spirit of singularity theory (see for instance [44]). Non-smoothness of the cost function implies the presence of inactivation along optimal trajectories. This principle becomes a regular theorem under the two following hypotheses: (1) the strict convexity of the cost function; and (2) the change of sign of the optimal control. Although technical, the convexity hypothesis is reasonable since: (a) most of the cost functions considered in the literature are actually strictly convex; (b) the set of strictly convex cost functions is very large; and (c) it ensures that what is minimized has a unique minimum. The change of sign assumption is clearly necessary (and actually observed), during fast point-to-point movements: indeed, after the agonistic muscles have been activated to accelerate the limb toward the target, they have to be deactivated in mid-flight and the antagonistic muscles activated in turn, to brake the movement.

Notably, this theoretical result is also valid for much more detailed models, which take into account viscoelastic properties of the muscular system and which specify the terminal equilibrium signals (e.g., muscle forces that compensate for elastic and gravitational forces, as in [45]).

In particular, the Inactivation Principle applies in two important cases. Firstly, it holds when considering that the net torque actually comes from agonistic and antagonistic torques. The result is that both torques are zero during the inactivation period. Secondly, this principle also holds when assuming that the torques are produced by muscles with non-zero response times, i.e., when the torques cannot immediately reach their maximum value. For instance, when the control is the derivative of torques (called gradient constraints case) or when the dynamics of muscles is modeled, the inactivation period is still present for fast movements minimizing the cost given in Equation 4.

These results are crucial for interpreting the inactivation on net torques as simultaneous inactivation of both agonistic and antagonistic muscles in practice.

A reciprocal question is whether partial or total inactivation could be predicted by other kinds of cost functions, notably by the smooth cost functions of the minimum jerk or torque change models.

Thus, does the presence of such periods of inactivation along optimal trajectories determine specific properties of the cost function?

In answer to this question, the following proposition is demonstrated:

***Necessity of non-smoothness.*** *If some optimal trajectories contain inactivation, then the term f in* *Equation 3* *cannot be smooth w.r.t. u at u* = 0.

This necessity of non-smoothness is stated in mathematical terms in the Mathematical Theory Subsection and the proof is given in Supporting Information (Text S1).

More precisely, it can be shown without any special assumption on the system (Σ), that the occurrence of total inactivation implies the generic non-smoothness of cost functions given in Equation 3. For partial inactivation, the set of terms *f* must be restricted to an open set of cost functions, strictly convex with respect to *u*. However, the set of strictly convex functions is very large and contains most of the cost functions from the literature.

#### Optimal solutions

Simulated movements, minimizing the compromise *Aw*/*Ae*, are depicted below and illustrate the theoretical results.

**Figure 1:**
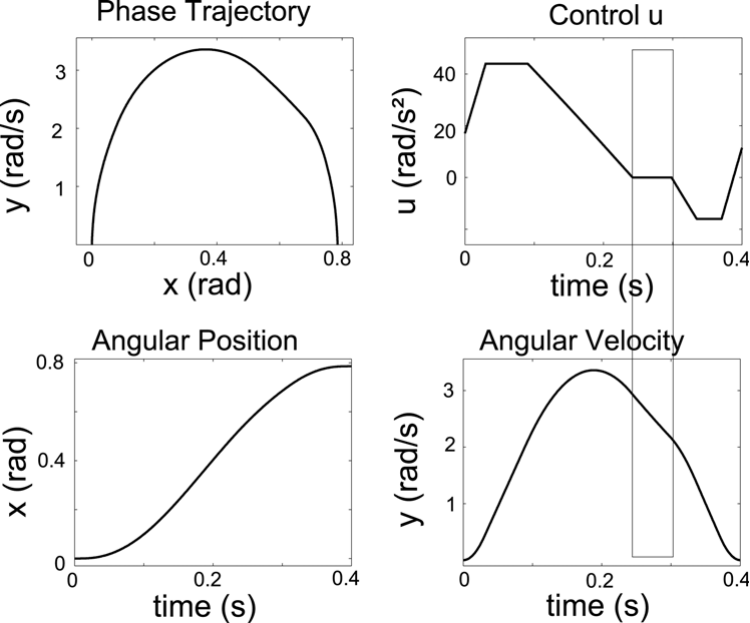
Results for a simulated 1-dof upward movement, with gradient constraints on the torque. The theoretical phase of inactivation of the muscles is shown (rectangular frame). Note that the time to peak velocity (TPV) is 0.47 in this case. It would be equal to 0.53 for the corresponding downward movement, according to experimental findings showing the same directional asymmetries. The signal *u* corresponds to the ratio between the net torque acting at shoulder joint and the arm's moment of inertia.

A simulated 1-dof movement minimizing the cost in Equation 4 is shown in Figure 1. In this example, bounds on the net torque and its derivative are imposed, forming a gradient constraint. Adding such a constraint allows us to control the derivative of joint torques in order to get smoother motor patterns, i.e., speed profiles with zero-acceleration at the initial and terminal times.

Notably, two important results hold in all instances of the model.

Firstly, in accordance with the Inactivation Principle, an *inactivation* period is observed slightly after the time of peak velocity during an upward movement (emphasized by a rectangular frame in Figure 1). During inactivation, the net torque acting at the shoulder is zero.

Secondly, speed profile is *asymmetric*, i.e., for an upward movement, the acceleration duration is shorter than the deceleration duration.

Although not illustrated, similar features appear during downward movements: the inactivation occurs slightly before the time of peak velocity, and more time is spent to accelerate the movement than to brake it.

**Figure 2:**
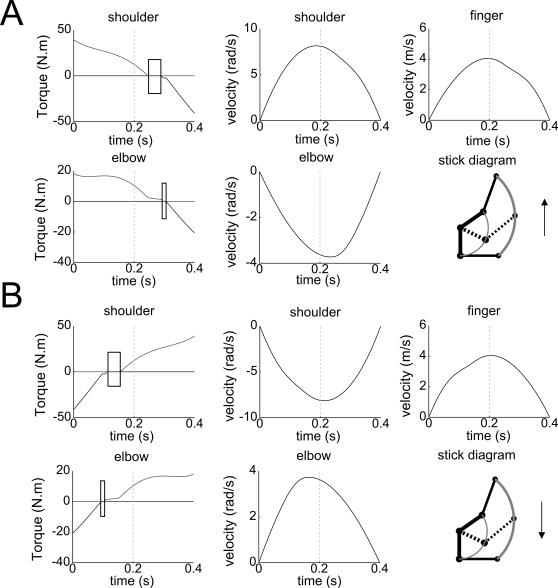
Results for a simulated 2-dof arm movement. (A) Upward direction. (B) Downward direction. Torques and angular velocities, respectively noted *u* (N.m) and *y* (rad/s), are plotted with respect to time (seconds), along with the finger velocity (m/s). The successive inactivation periods at each joint and the asymmetries of the velocity profiles are clearly visible.

Simulated 2-dof vertical arm movements are also depicted in Figure 2. Partial inactivation, illustrating the Inactivation Principle, occurs at each joint separately (elbow and shoulder). Moreover, fingertip velocity profiles are asymmetric during upward and downward movements, as for the 1-dof case. Since the response time of muscles was not modeled in this case, jumps on the joint torques occur at the initial and final times, leading to non-zero accelerations on the corresponding velocity profiles.

### Experimental Verification

Although human vertical arm movements are studied here, the above theoretical results may apply to locomotion, whole-body reaching, and more generally to any mechanical system described in the Mathematical Theory Subsection.

Firstly, we show that minimizing the compromise *Aw*/*Ae* is consistent with temporal and spatial features of biological arm movements. Secondly, we report simultaneous inactivation of agonistic and antagonistic muscles during arm movements. This suggests that the proposed criterion is also relevant at the muscular level and gives insights concerning the cost minimized during fast arm movements.

#### Kinematic level analysis

**Figure 3:**
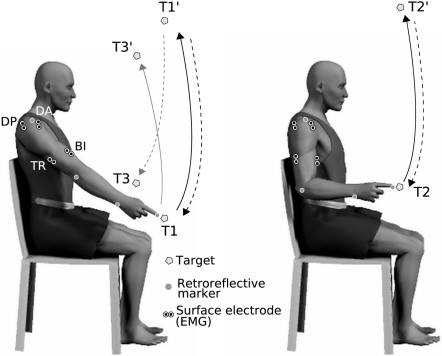
Illustration of the experimental setup. (*Left*) Black trajectories show the 1-dof pointing task between targets T1 and T1′. Gray trajectories show the 3-dof experiment, starting from fully-extended arm postures (targets T1-T3′ and T1′-T3). (*Right*) Vertical 2-dof pointing movements, between targets T2-T2′. The position of the surface electrodes (for EMGs) and the kinematic markers is shown. Abbreviations: DA, Deltoid (Anterior); DP, Deltoid (Posterior); BI, Biceps and TR, Triceps.

**Figure 4:**
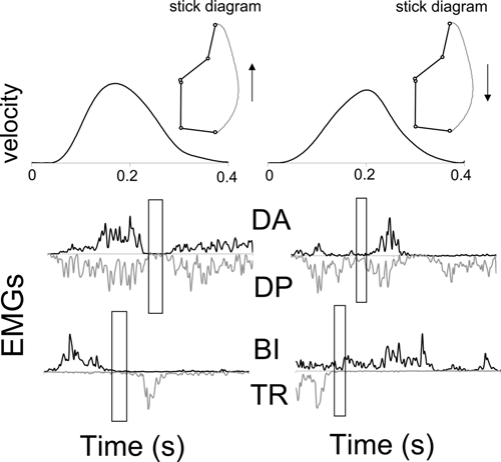
Typical experimental data of a 2-dof arm movement performed in upward (left) and downward (right) directions. Finger velocity profiles (upper part) and four EMGs (lower part) are amplitude normalized. The periods of muscular inactivation are emphasized by means of rectangular frames. The same abbreviations as in Figure 3 are used.

In previous works [35],[36], during upward and downward arm movements performed in the sagittal plane, fingertip velocity profiles showed asymmetries depending on movement direction and speed, and fingertip paths were slightly curved. For 2-dof vertical arm movements (targets T2-T2′, see Figure 3), movement duration (MD) was equal to 0.43±0.05 s. The relative time to peak velocity (TPV) was equal to 0.42±0.02 and 0.53±0.04 for upward (U) and downward (D) directions respectively. These asymmetries were significant (t-tests, *p*<0.001). Figure 4 (upper row) illustrates typical tangential velocity profiles of fingertip motion.

Simulations by means of the model proposed in the present study were consistent with these experimental results (see Figure 2), since TPV is 0.46 and 0.54 for U and D directions, respectively.

Typical fingertip paths can be observed on the stick diagrams (depicted in Figure 4). Fingertip paths were curved: average fingertip path curvature (FPC) was equal to 0.14±0.04. These values were close to those (0.20) simulated by means of the model.

**Figure 5:**
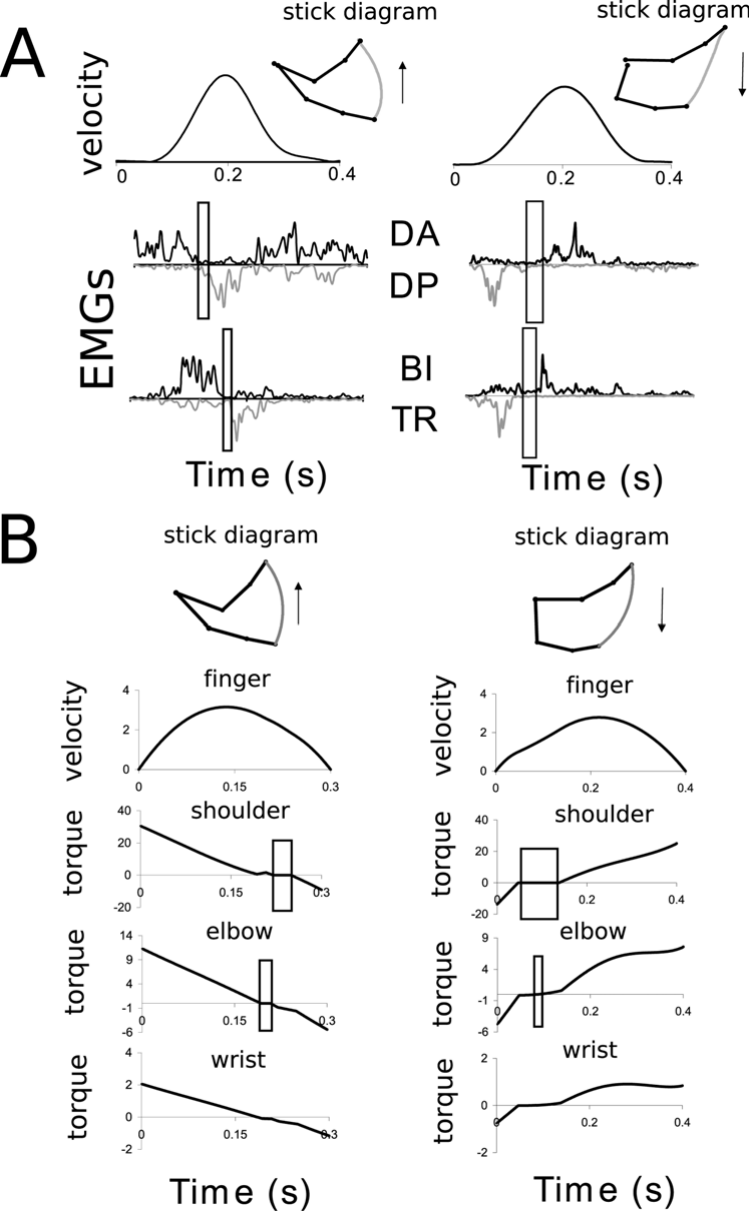
Typical experimental data of a 3-dof vertical arm motion performed in upward (left) and downward (right) directions. (A) Experimental results. Finger velocity profiles (upper part) and four electromyographic signals (lower part) are amplitude normalized. (B) Simulated results. The shoulder, elbow and wrist joints were free to move. Torques and velocity are given in N.m and m/s, respectively. The solutions were computed using Pontryagin's Maximum Principle (as for the 2-dof case depicted in the Materials and Methods Section, but with more complicated formulae). Moreover, the transversality conditions of Pontryagin's Maximum Principle were necessary since the location of the target in task-space led to a set of possible terminal postures, given by a 1-dimensional manifold. The periods of muscular inactivation are emphasized by means of rectangular frames. The same abbreviations as in Figure 3 are used.

Figure 5 illustrates typical 3-dof arm movements (targets T3-T1′ and targets T1-T3′). This experiment was designed to test the influence of the initial arm configuration upon finger kinematics as well as the influence of movement direction (U versus D) upon final arm posture. Indeed, in a redundant system such as a 3-dof arm movement, the CNS must select the final posture of the arm among an infinite number of possibilities. The MD recorded in this condition was on average 0.38±0.06 s, and finger kinematics, as in the experiments described above, were significantly asymmetric (*p*<0.001) with respect to the movement direction (U: FPC = 0.13±0.03, TPV = 0.47±0.02; D: FPC = 0.09±0.03, TPV = 0.51±0.02). The simulated movements fitted quite well with those recorded in practice (U: FPC = 0.15, TPV = 0.46; D: FPC = 0.14, TPV = 0.53). Moreover, the simulated final arm postures (wrist: 14°, elbow: 68°, shoulder: −23° for U and wrist: 25°, elbow: 74°, shoulder: −88° for D) were similar to those measured experimentally (wrist: 19±3°, elbow: 63±4°, shoulder: −25±3° for U and wrist: 20±3°, elbow: 90±5°, shoulder: −99±5° for D).

Thus, the proposed optimality criterion seems to be well suited for the planning of redundant vertical arm movements.

Interestingly, optimizing the compromise *Aw*/*Ae* allows us to reproduce the kinematic asymmetries observed in vertical arm movements. However, this does not prove whether these directional asymmetries are caused by gravity, inertia, or both. Indeed, according to some authors, the difference in initial arm configurations between upward and downward movements would determine different inertial interactions between the upper arm and the forearm, which would in turn cause the observed asymmetries [19],[26].

Nevertheless, similar directional asymmetries were observed during 1-dof movements (i.e., fully-extended arm) performed in the sagittal plane, while the distribution of the masses around the shoulder joint remained approximately constant [37],[38].

**Figure 6:**
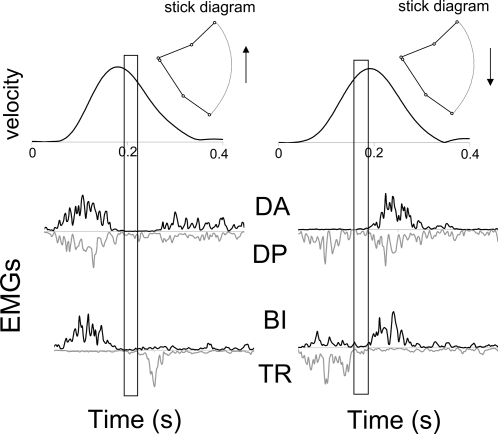
Typical experimental data of a 1-dof arm motion performed in upward (left) and in downward (right) directions. Finger velocity profiles (upper part) and four electromyographic signals (lower part) are reported. Note the asymmetries of the speed profiles and the simultaneous inactivation of all muscles which occurs near the velocity peak. Data are amplitude normalized and the horizontal axis denotes time (in seconds). Same abbreviations than in Figure 3. The same abbreviations as in Figure 3 are used.

In this 1-dof case, arm kinematic features in the sagittal plane were well explained by the model. The MD recorded in this condition was on average 0.36±0.04 s. Since the fingertip path was necessarily a circular arc, the TPV was the only significant measure. The experimental results confirmed those of previous studies (see Figure 6). The TPV parameter was significantly smaller for upward than downward movements (0.42±0.02 versus 0.54±0.04, respectively, *p*<0.001). In accordance with this, simulations by means of the proposed model predicted smaller TPV values for arm movements performed against gravity compared to movements performed with gravity (0.47 versus 0.53, respectively).

Moreover, this asymmetry did not result from the additional term *Ae* but from the absolute work term *Aw*. Indeed, the minimization of the absolute work alone (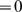 in Equation 4) in the 1-dof case inherently led to lower TPV values of upward compared to downward movements. Interestingly, the difference between U and D movements in the gravity field was caused by the zero-torque period, during which the arm is approximately in free fall.

#### Muscular level analysis

In previous studies [35],[36], during vertical arm movements performed at slow speeds (movement durations longer than 0.7 s), only flexor muscles were active: mainly the anterior deltoid, which initiated the action during upward movements or braked the action during downward movements. However, at fast speeds (movement durations shorter than 0.7 s), extensor muscles were also active, since gravity alone was not sufficient to accelerate downward and decelerate upward movements.

Here, simultaneous inactivity of muscles during rapid arm movements, near the time of peak velocity of the fingertip, was specially examined, to check the Inactivation Principle. From an experimental point of view, silent phases should simultaneously appear on the EMG signals of opposing muscles, if the proposed cost function is relevant at the muscular level (this is related to the direct optimal control approach). Conversely, if such an inactivation is checked, then, under the assumption that motor planning minimizes a certain integral cost, one can conclude that this cost contains a term similar to the absolute work. Thus, the presence of inactivation will imply certain properties of the cost function (this is related to the inverse optimal control approach).

Before considering new results, it is worthy to note that, in accordance with the theoretical predictions, simultaneous inactivation may not appear in practice if movements are too slow, too small, or involve muscles with large response times. However, the appearance of inactivation is a phenomenon theoretically independent of the following factors: gravity and number of degrees of freedom of the motion.

The presence of inactivation periods was first investigated by measuring EMG signals of different muscles during rapid pointing movements performed with the arm fully extended (1-dof case). Figure 6 shows typical experimental results.

The first and second columns show upward and downward movements, respectively. Muscle silent phases are noticeable in this figure (emphasized by a rectangular frame), in agreement with the theory. The main flexor and extensor muscles acting on the shoulder joint are simultaneously inactive, so that the net torque resulting from their actions is almost zero during this short period.

For upward movements, simultaneous inactivation of all muscles appeared clearly during a short time interval in the second half of the motion. In some trials, the triceps remained slightly contracted, thus actively maintaining the arm fully extended. For downward movements, an inactivation also appeared, although less clearly, during the first half of the movement. This simultaneous inactivation of all muscles lasted on average for approximately 30 ms and was clearly observed in 85% of trials, for upward movements. During this period the arm was almost in free fall, an energetically costless movement. Notably, the activities of all muscles stopped at the same instant. This synchronization suggests that muscle inactivation results from an active optimal motor strategy. Taking into account the electromechanical delay which elapses between the muscle bundle depolarization and the actual force production, this period of inactivation appeared as was expected from the theory (i.e., slightly before and after the maximum velocity for upward and downward movements, respectively).

A typical muscular pattern for the vertical 2-dof case is depicted in Figure 4. Here also, simultaneous inactivation of pairs of muscles acting on each joint occurred. Notice the lag between the inactivation at the elbow joint and at the shoulder joint, illustrating that in the 2-dof case the inactivation occurred at each joint separately. This is in agreement with the corresponding numerical simulations (see Figure 2) and the theoretical results concerning partial inactivation.

The appearance of simultaneous inactivation was also checked in movements starting from different initial arm configurations (i.e., starting from various initial arm postures; targets T1-T3′ and targets T1′-T3).

For both upward and downward movements, this inactivation phenomenon is shown in Figure 5, where muscular activities and simulated net torques can be compared.

To summarize, for the set of movements and conditions tested, both movement kinematics and muscles activities confirm the relevancy of the theoretical model.

## Discussion

Limb movement planning theory, presented in this study, focuses on fast, open-loop, vertical arm movements, and is based upon the assumption that such movements are optimal with respect to a certain integral cost. Within this framework, the question was to characterize possible cost functions.

### Direct Optimal Control

A model that minimizes a cost based upon the absolute work (i.e., an energetic optimality criterion) has been shown to allow simulating plausible arm movements in the sagittal plane. This was checked by means of three relevant kinematic features: fingertip path curvature, asymmetry of fingertip velocity profiles, and final arm posture.

Since this cost function is non-smooth, the Inactivation Principle can be stated: for a large class of non-smooth cost functions, the net torque acting on a joint is zero during a short period occurring around the mid-path movements that are sufficiently rapid. This principle is also valid if a pair of agonistic-antagonistic actuators is considered, exerting opposite torques. Each of the torques is zero during an inactivation period which still appears if the biomechanics of the muscles is considered, when response times are brief (a few tens of milliseconds). For longer response times, complete inactivation is progressively replaced by low-levels of muscular activities.

Such quiet periods in the EMGs of opposing muscles were observed during fast arm movements (see Figures 4, 5, and 6), which suggests that this optimality criterion is suitable.

The suitability of a similar non-smooth cost function was also found for animals in a recent study [46]. The author concludes that the locomotor pattern of legged animals is optimized with respect to an energetic cost based upon the “positive work” of forces.

However, the direct optimal control approach does not prove that the motor planning process actually minimizes energy expenditure. It just shows that such a criterion is plausible because it provides realistic behavior. Indeed, several other cost functions or theories may lead to similar results.

For instance, muscle inactivation was also interpreted as a consequence of the Equilibrium Point hypothesis [47]. According to this interpretation, the threshold position control and the principle of minimal interaction would, together, determine the “Global EMG minima” which appear simultaneously in all muscles during rhythmic movements, near the point of direction reversals. Nevertheless, in the theory proposed here, inactivation is somewhat different: it appears near the time of peak velocity, and the precise interval of inactivity may be different at different joints. Moreover, inactivation is still predicted even if biomechanics of muscles, inertia and external forces are taken into account, which is not the case in Equilibrium Point theory [47].

Alternatively, it could be also considered that the CNS simply activates and deactivates the muscles, explicitly determining inactivation phases. However, this would be an argument against our main assumption that the brain tries to minimize some costs. Here, under this assumption, inactivation provides information on the cost function.

### Inverse Optimal Control

The theoretical results also allow us to characterize the non-smoothness of the cost function once the simultaneous inactivation of opposing muscles is measured in practice, during movements presumed as optimal.

Using mathematical transversality arguments from differential topology we proved that the minimization of an absolute-work-like cost during arm movements is a necessary condition to obtain inactivation phases along optimal trajectories. In other words, assuming that human movements are optimal with respect to a certain integral cost, the simultaneous inactivation of muscles that we observed provides evidence for an absolute-work-like cost.

Notably, this simultaneous inactivation of opposing muscles, which is a singular phenomenon, cannot be predicted by models using smooth cost functions, such as the minimum endpoint variance [27], the minimum jerk [16], or the minimum torque-change [17]. Those models would predict deviations from “zero torque”, whereas singularity analysis proves the existence of an exact inactivation period.

Simultaneous inactivation periods also appeared on intra-muscular EMG traces recorded from monkeys when performing horizontal arm movements (see Figure 5 in [48]). These findings suggest that the minimization of the energy expenditure may be a basic motor principle for both humans and animals.

It should be emphasized that such an equivalence between specific movement features and well-identified properties of the cost function is not common in studies using optimal control approach for movement planning.

### Validity of the Model

The simulated movements replicated the experimental records accurately, except, obviously, for the bang-bang command signals which provide non-zero accelerations at the beginning and end of the movement (see Figure 5). The patterns of motor command are actually smoothed by the biomechanical characteristics (low-pass filters) of the muscles. As pointed out by several authors some models have been rejected hastily due to the lack of biological validity of their optimal solutions (bang-bang behaviors) [15],[49]. This problem was also discussed in a study where the authors used a similar non-smooth cost function based upon the “positive work” of forces [23]. They noticed that the abrupt velocity profiles predicted by their model were non-realistic but might actually be smoothed by modeling muscles dynamics. In fact, depending on the precision of modeling, different conclusions may be drawn. This is illustrated in Figure 1 where gradient constraints on the torques lead to smoother motor patterns whereas Figure 10 shows solutions in a simpler case of torque control. In the first case the acceleration is continuous while in the second case the acceleration jumps at the initial and final times (to make the transition between posture and movement). Nevertheless, in both cases, inactivation is present and fingertip velocity profiles reproduce the experimental directional asymmetries. Thus, these relevant features of movements are not affected by such changes in modeling. The reason for not systematically considering more precise levels of modeling is twofold. Firstly, it causes important additional computational difficulties, and secondly, many more parameters, which are not always well-known, appear in the model.

Here, the model depends on a few parameters. Firstly, the maximum torque that can be developed by each muscle is finite. In particular, this determines the shortest possible movement duration in order to complete the pointing task. Nevertheless these maximum torques did not seem to be reached in practice (at least during the movements tested here) so that their precise values were not important for the present study. Secondly, the weighting parameters that appear in the cost could depend on the individual and the task goal. However, they are not critical with respect to the qualitative behavior of the optimal solutions and, although their values could be discussed, the simulations obtained using this model were accurate for a large range of these parameters. Importantly, the whole theory holds without precise constraints on these parameters. A first example is given by the strongly consistent kinematic difference in the 1-dof case for movements performed in the upward versus the downward direction. For instance, for an upward movement (1-dof, 45° and 400 ms), the relative time to peak velocity (TPV) ranged between 0.43 and 0.5 for weighting parameters ranging between 0 and 10. For the corresponding downward movement, TPV ranged between 0.57 and 0.5. The classical models [16]–[18] were not able to reproduce this directional difference in the speed profiles observed in vertical arm movement executed with 1-dof [37]. Moreover, it has been found that this difference disappeared for movements performed in the horizontal plane, either in upright or reclined postures [37],[38]. This behavior is experimentally well established and can be easily verified with simulations. Interestingly, it is predicted by our optimality criterion, whatever the choice of the tuning parameters. A second example concerns the final posture selected by the model. The exact terminal limb configuration depends on these weighting parameters. However, we tested several instances of the model, for weighting parameters ranging between 0.05 and 1. In all instances, the simulated terminal postures were in the range of those measured in practice.

In order to check the validity of the present model, its predictions were also compared with well-known experimental findings, without trying to fit the data. The tuning parameters used are defined in the Materials and Methods Section.

Movement curvature is known to depend on movement duration [36],[50]. Here, the 2-dof model predicts a change in the fingertip path curvature (FPC) when movement duration varies. For the movements tested in Figure 2, the FPC ranged between 0.18 and 0.23 for movement durations of between 0.2 s and 1 s.

Moreover, the final postures have been found to be invariant with respect to the speed of the movement [8] and to the addition of a mass of 600 g on the forearm [9]. Here, in the 3-dof case, the final posture does not significantly vary with movement duration. For instance, the final postures changed by less than 3° (maximum change at each joint) while the movement duration ranged between 0.2 s and 1 s (tested for U and D movements that appeared in the left column of Figure 5). Also, adding a mass of 600 g to the forearm did not change the simulated final limb configuration: the model predicted less than 0.5° of variation at each joint.

In the proposed model, the final posture is selected as the final limb configuration that minimizes the amount of the compromise *Aw*/*Ae* necessary to bring the finger to the target. Movements directed toward a single target were tested for various starting configurations of the arm. It resulted in changes in the final posture (about 1°, 10°, and 15° of variability at the shoulder, the elbow, and the wrist levels, respectively). Thus, the final posture depends on the initial configuration of the arm, in agreement with experimental results [21].

**Figure 7:**
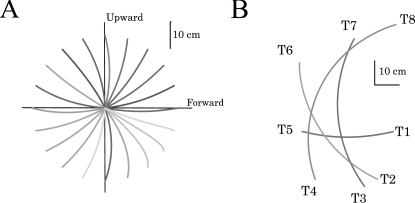
Simulated fingertip paths in the 2-dof case. (A) Finger trajectories for different movements toward targets located on a circle. Initially the finger position is at the center of the circle. For more details about the task and to compare the results, see [33],[52]. (B) Finger trajectories for four different movements performed in the sagittal plane (T1 to T5, T2 to T6, T3 to T7, and T4 to T8). For more details about the task and to compare the results, see [6].

It must be noted that the minimum torque-change and the minimum force-change models failed to predict the curvature of movements when antigravity torques were implied in the optimization process, according to Figure 3 in [33]. In contrast, the finger trajectory for a 2-dof arm predicted by our model (for the same movements of duration equal to 400 ms) was quite realistic (Figure 7A). This was also in agreement with the experimental finger paths observed in Figure 4 in [6] for other movements performed in the sagittal plane (see Figure 7B).

Although the proposed model was only tested in a sagittal workspace, it appears to be well-suited for a large set of movements and may, thus, motivate future extensions of the model to 3-dimensional movements.

### Integration of Gravity

Several investigators have proposed that the CNS optimizes inertial forces and compensates gravitational forces at each instant [19],[26]. Static and dynamic forces were assumed to be controlled separately. Although plausible, this idea is hardly compatible with several experimental results. For instance, when considering an upward movement in the sagittal plane performed with the arm fully-extended (1-dof case), according to such a viewpoint, agonistic (anti-gravitational) muscles should be active throughout the movement (corresponding to a tonic component of EMGs) [31]. In this case, a muscular activity counteracting the gravity would be necessary to continuously maintain the arm, as if it were at equilibrium at each instant, and would be noticeable in EMGs. However, EMG recordings showed that the activities of the agonistic muscles were quasi-null near the time of peak velocity suggesting, thus, that no muscle was acting against gravity at this instant. Moreover, it may explain why, after subtracting the tonic activity from rectified EMG data, some authors obtained negative phasic activities of some muscles (e.g., see [51],[52]). Rather than resulting from errors in the evaluation of the tonic component of muscles activity, the gravitational and inertial forces could just be integrated into the same motor plan, within the minimization of energy expenditure. In that case, an explicit separation between tonic and phasic activities of muscles could be impossible, at least for fast movements.

It must be noted that separating static and dynamic forces is not the same as separating posture and movement. Indeed, static and dynamic forces are present during posture maintaining. Neuro-anatomical and experimental evidences for distinct controls of posture and movement were reported in [53]. Thus, the present results concerning inactivation do not contradict the hypothesis that, while maintaining posture, anti-gravity control seems to be tightly related to the muscular system's viscoelastic properties (see [54] for a study of equilibrium control during quiet standing). This problem was not addressed here since we focused on the control of the transient phase of fast movements.

### Conclusions

In conclusion, from a methodological point of view, the novelty of the present work is to introduce a hypothetical-deductive approach in studies focusing on motor planning of arm movements. The possible existence of the inactivation phenomenon was deduced from a mathematical analysis which aimed to reproduce directional asymmetries in arm movements performed in the sagittal plane. Then, the presence of these inactivation periods produced by the model was confirmed by the EMG signals obtained from experimental data. The mathematical analysis showed that this inactivation was a necessary and sufficient condition for the minimization of an absolute-work-like cost. As far as we know, this is the first time that such a condition has been proved in studies investigating optimality principles in human movement. These results suggest that, considering that inactivation is a short and quite singular phenomenon, more attention should be paid to this specific movement feature in future studies.

Two major conclusions can be drawn:

1. Both inertial forces (necessary to accelerate movements) and gravitational forces (acting on the limbs) appear to be integrated in motor planning within the minimization of an absolute-work-like cost.
2. The connectivity of the command circuits and the signals that they process should result in synchronized periods of muscles inactivation.

## Materials and Methods

### Experimental Procedures

#### Participants

Six male participants (mean age 29.6±8.9) volunteered to participate in the experiment. All were healthy, right-handed, and with normal or corrected-to-normal vision. The experimental protocol used was in accordance with the principles expressed in the Declaration of Helsinki.

#### Motor tasks

From a sitting position, participants performed 1-dof (shoulder rotation), 2-dof (shoulder and elbow rotations), and 3-dof (shoulder, elbow and wrist rotations) pointing movements in the sagittal plane. The experimental apparatus and the pointing movements are illustrated in the Figure 3. In all experimental conditions, participants were instructed to execute visually-guided, fast arm movements towards the targets without final correction (here denoted *Ti* or *Ti*′, *i* = 1..3, and that consisted of a small sphere of 5 mm in diameter). The duration of these movements was about 0.4 s. In order to familiarize themselves with the motor tasks and the experimental apparatus, they were trained (5 movements in each experimental condition) by means of a metronome set at 0.4 s. During the experiments, a single data acquisition file consisted of an upward-downward sequence of pointing movements between paired targets. A significant pause (>1 second) was requested between two pointing movements. Participants performed 10 trials in each condition (i.e., a total of 60 pointing movements per participant). After data analysis, all pointing movement durations were found to range between 0.3 s and 0.5 s and the final precision was similar (error less than 3 cm) between conditions. Thus, all participants were considered to have successfully performed the requested tasks.

***Single-joint arm pointing (targets T1-T1′).*** The two targets were placed in the sagittal plane (shoulder abduction equal to 0°) and symmetrically (40° above and below) from the participants' right shoulder joint. The participants performed upward and downward pointing movements (amplitude: 80°), with the arm fully extended (i.e., rotation around the shoulder joint only). Movements started either from an upward or downward position (50%). Note, that participants' elbow and wrist joints were motionless during this experiment.

***Two-degree of freedom arm pointing (targets T2-T2′).*** The initial configuration of the arm, for the target T2, was the following: shoulder 0° flexion and 0° abduction; elbow 90° flexion and 90° pronation. The two targets (inter-target distance: 90 cm) were placed symmetrically in the sagittal plane (45 cm above and below) from the participants' right shoulder joint. The horizontal distance of the lower target from the participants' right shoulder joint corresponded to the length of the forearm-wrist-finger horizontal alignment. Movements started either from an upward or downward position (50%). In this condition, the wrist was artificially immobilized by means of straps.

***Three-degree of freedom arm pointing (targets T1-T3′ and targets T1′-T3).*** The participants were asked to start from a fully-extended arm position (in the sagittal plane, shoulder abduction equal to 0°) and to reach a target placed in a position such that an elbow flexion was necessary, in addition to a shoulder joint rotation (see gray trajectories in Figure 3). In this condition, the wrist was free to move. The target T3 was placed with respect to the target T1 (15 cm backward and 15 cm upward). The inter-target distance was 70 cm. The target T3′ was placed symmetrically with respect to the target T1′. Movements started either from an upward or downward position (50%).

#### Material

The system used to capture arm movements was an optoelectronic device (SMART-BTS, Milan, Italy). Nine cameras were used to capture the movement of four retro reflective markers (15 mm in diameter), placed at well-defined anatomical locations on the right side of the body (acromial process, humeral lateral condyle, ulnar styloid process, and the apex of the index finger). Surface electrodes which captured muscular activity were placed on the following muscles: the biceps, the triceps, the anterior deltoid, and the posterior deltoid (see Figure 3 for an illustration of the placement of electrodes and markers). Two silver-chloride surface electrodes of 10-mm diameter were positioned on the belly of the muscle (with the skin previously shaved and cleaned) with an inter-electrode distance (center to center) of 2 cm. The reference electrode was placed on the left ankle. The placement of surface electrodes was then checked by asking subjects to produce isometric contractions at each joint and in various directions. Sampling frequencies were 120 Hz and 960 Hz for kinematics and EMGs, respectively.

#### Data processing

Data processing was performed using custom software written in Matlab (Mathworks, Natick, MA). Recorded kinematic signals were low-pass filtered using a digital fifth-order Butterworth filter at a cut-off frequency of 10 Hz. Finger movement onset was defined as the moment at which linear tangential velocity of the index fingertip exceeded 5% of its peak and the end of movement as the point at which the same velocity dropped below the 5% threshold. Movement duration (MD) was defined as the time-interval between the onset and the offset times.

The following kinematic parameters were then calculated: the relative time to peak velocity (TPV), defined as the ratio of acceleration duration to total movement duration, and the fingertip path curvature (FPC), defined as the ratio of maximum path deviation from a straight line connecting the initial and the final points of the trajectory. Both FPC and TPV parameters were often considered as relevant indices for the planning of arm movements [36],[37],[55].

Stick diagrams were also reconstructed to depict the initial and final arm configurations in the vertical plane.

EMG data were band-pass filtered (20–400 Hz). The root mean square (RMS) of EMG data was computed over 5 ms intervals. The electromechanical delay was evaluated by synchronizing the first agonistic onset time with the onset time of the fingertip. The onset time of an EMG burst was defined as the moment at which the smoothed RMS signal (low-pass filtered at 5 Hz) exceeded 10% of its peak. A muscle was considered as inactive when the corresponding RMS was below 10% of its maximum value. Individual, rather than averaging, EMG inspections were performed because of the briefness of the phenomenon searched for.

#### Statistical analysis

All variables (i.e., MD, TPV and FPC) were normally distributed (Shapiro-Wilk's test) and their variance was equivalent (Levene's test). Statistical comparisons were performed by means of paired t-tests.

#### Simulations

Simulations were performed using custom software written in Maple (Maplesoft, Waterloo, ON) for the formal calculations and in Matlab for the numerical computations. The optimal solutions were actually found by adjusting the “adjoint vector” (see next section) by means of the fsolve Matlab function (Gauss-Newton method).

### The Mathematical Theory

This section is devoted to technical details and proofs of the results presented in the Theoretical Analysis Subsection. It is organized as follows.

Firstly, we present the general setting of the optimal control problem under consideration. Secondly, we present the examples that will be used to illustrate the theory. After presenting some prerequisites that may be helpful to understand the main mathematical results, we state two theorems concerning the Inactivation Principle and the necessity of non-smoothness. Then, some details on the computation of the optimal solutions using Pontryagin's Maximum Principle [41] are reported (for the 1-dof and 2-dof cases). Finally, three extensions of the model are given in the case of i) gradient constraints on the control; ii) distinct control of agonistic and antagonistic torques; and iii) modeling the dynamics of agonistic and antagonistic muscles.

#### The general setting and the optimal control problem

We consider mechanical systems with generalized coordinates 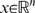 and Lagrangian:
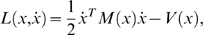
where *M*(*x*) is the inertia matrix (which we assume to be symmetric and invertible) and *V*(*x*) is the potential energy (here due to gravity).

We divide the external generalized forces acting on the system into two components: the first one, denoted by *τ* = *S*(*x*)*u*, resulting from the input *u* and the second one, denoted by *N*(*x*, *x* ˙) representing any other forces acting on the system, mainly friction forces.

We assume that the control acts on every degree of freedom, that is, 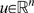 and *S*(*x*) is invertible. Moreover, in the exactly-fully-actuated case that we consider first, we assume to directly control each degree of freedom, that is *S*(*x*) = *Id*. This is assumption is always verified up to some feedback. Indeed, we can always add a “feedback pre-compensator” of the type *τ* = *S*(*x*)*u*. From a theoretical point of view it is just a change of variable. From a practical point of view, it requires the knowledge of the state *x* of the system, or some estimation of it.

The equations of motion are given by substituting the value of *L* into Lagrange's equation,
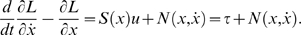
They are exactly of the form given by Equation 1, with
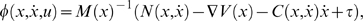
(5)where the Coriolis matrix 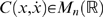 is defined as:
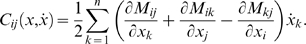



Then, in order to get the control system, we set *X* = (*x*, *y*) = (*x*, *x* ˙) and rewrite the system as:
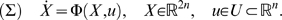



We can also write the equations of motion in the Hamiltonian formalism.

We define the Legendre transform: (*x*, *x* ˙)↦(*x*, *p*), by 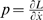, and we introduce the Hamiltonian *h*(*x*,*p*) of the problem:
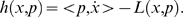
Then, we get the equations of the motion via the characteristic field of the Hamilton-Jacobi equation:
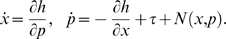
As a consequence, the work *w* of external forces, *w* = ∫(*τ*+*N*(*x*,*p*))*dx* is identically equal to the variation of the Hamiltonian:
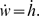
In particular, if there is no friction (*N* = 0), the variation of the Hamiltonian is equal to the work of controlled forces *τ* during the motion.

Thus, the work of controlled forces is:
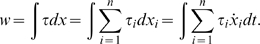
Here, the work of controlled forces is counted algebraically: a motion in one direction followed by a motion in the opposite direction may give zero work.

In the following, we will consider the absolute work *Aw* of controlled forces, which corresponds to the energy spent to control the system:
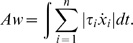
(6)


In coordinates *X* = (*x*,*y*), *A* ˙*w* is the function:
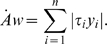



The cost we will minimize is a compromise of the form:
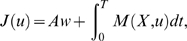
(7)in which *M*(*X*,*u*) is a comfort term that for technical reasons we will assume to be smooth and strictly convex w.r.t. the control *u* (**assumption A**).

*Remark 3.* (1) More generally we could consider an integral cost of the form:
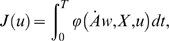
(8)

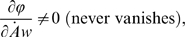
(9)where *ϕ* is smooth and *ϕ*(*A* ˙*w*(*u*), *X*, *u*) is strictly convex w.r.t. *u* (2) The assumption of strict convexity, although technical, is natural: it implies that the function *ϕ* has a unique minimum with respect to *u*. The weakest possible hypothesis to obtain the Inactivation Principle (see Theorem 2) is precisely that *ϕ* has a unique minimum w.r.t. *u*. In that case, existence of a minimizing trajectory will not be guaranteed (it has to be assumed). Assuming strict convexity is a way to assume both a unique minimum w.r.t. *u* and the existence of a minimizing trajectory (see [43] for a precise proof of this last fact). (3) Due to the absolute work term, the proposed cost function is non-smooth (non-differentiable) w.r.t. *u* at *u* = 0. However it is Lipschitz-continuous at *u* = 0. This slight difference is important in our study. (4) In fact the typical non-smoothness (Lipschitz) is that of the absolute value function. But it can be easily taken into account the fact that “negative work” costs less than “positive work” (this last fact was stressed by a referee): in place of the function |*u*|, one has to consider the Lipschitz function *λ*|*u*| for *u*>0 and *μ*|*u*| for *u*≤0. We decided here to limit ourselves to the “non-weighted” absolute work, for the sake of simplicity in exposition.

We now define our optimal control problem. We consider the following controlled system (Σ):
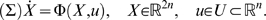



Fix a source point 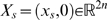, a target point 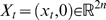 and a time *T*.

Then, the optimal control problem is:
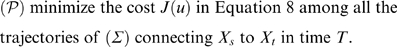



The following theorem proves that this problem is well-posed.

#### Theorem 1 (existence of optimal trajectories)

*The minimum is reached by some optimal trajectory.*

This is shown in [43] in the 1-dof case, and is a consequence of boundedness of the controls and convexity with respect to *u* of both the cost function and the system (Σ). The idea is that a minimizing sequence of trajectories converges for some compactness reason of Ascoli type, and the limit is a trajectory of the system by convexity. General results of this type may be found in [56].

#### The main examples

We will consider different examples of mechanical systems throughout the paper.

In all these examples, the cost is the compromise between the absolute work *Aw* and the acceleration energy *Ae*, i.e., a compromise of type given by Equation 7 with:
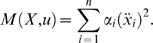
(10)


The parameters (*α_i_*)*_i_*_ = 1..*n*_ are strictly positive constants. This comfort term expresses the fact that sensorimotor system penalizes large accelerations (thanks to learning) in order to protect articulations and tendons. Such an optimality criterion was used in [15].

In the 1-dof case, this weighting parameter was set to 0.25. We set *α*_1_ = 0.25, *α*_2_ = 0.25 and *α*_1_ = 0.05, *α*_2_ = 0.1, and *α*_3_ = 0.25 in the 2-dof and 3-dof cases, respectively. Nevertheless, we also simulated movements with weighting parameters ranged between 0.05 and 1, and all these instances of the model lead to plausible movements. Therefore, these parameters may be considered as tuning parameters to improve the quantitative fitting of the model to each participant.

Note that this term *M*(*X*,*u*) is strictly convex with respect to *u* (in accordance with assumption A).

We will now consider the different mechanical systems describing vertical movements of an arm with 1-dof and 2-dof.

***Example 1. The one-degree of freedom arm.*** We consider a 1-dof arm moving in the vertical plane, in the gravity field, and without friction.

The control system is:
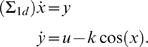
(11)


Here, the constant *k* reflects the action of the gravity field, 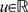 is the net torque acting at the joint, and *u* is bounded (*u* ^−^≤*u*≤*u* ^+^ with *u* ^−^<0<*u* ^+^).

***Example 2. The two-degree of freedom arm.*** We consider a 2-dof arm moving in the vertical plane, in the gravity field, and with friction forces.

**Figure 8:**
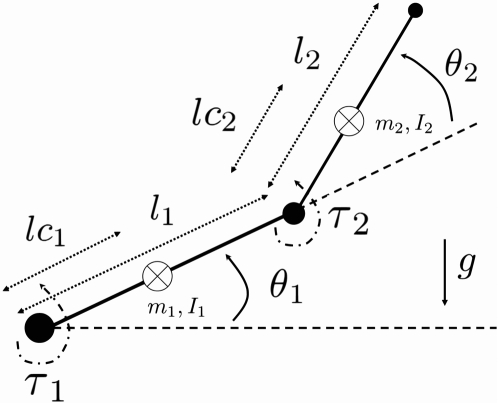
Mechanical model of the 2-dof human arm. The subscripts 1 and 2 denote the shoulder and elbow joints respectively. Generalized coordinates *θ*, joint torque *τ*, moment of inertia *I*, segment mass *m*, segment length to the center of mass *lc*, and gravity acceleration *g* are denoted.

The mechanical equation of the movement is:
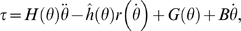
(12)in which *H* is the (symmetric positive definite) matrix of principal inertia moments, 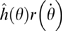 is the Coriolis term, *G* is the vector of gravitational torques, and *B* is the matrix of friction terms (a constant here). The term *τ* is the vector of external torques (the controls in our case), i.e., *τ* = *u*. We get (see also Figure 8):
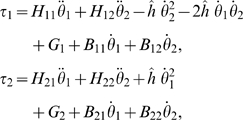
(13)with
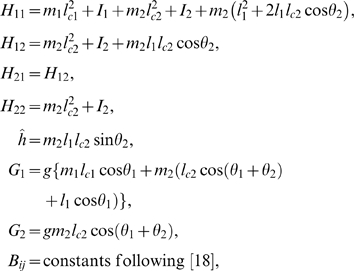
in which the following notations are set and the numerical values come from [57]:

*M_s_* total mass of the subject (kg),
*L_s_* height of the subject (m),
*m*_1_ mass of the arm (≈*M_s_*×0.028 kg),
*m*_2_ mass of the forearm (+hand) (≈*M_s_*×0.022 kg),
*l*_1_ length of the arm (≈0.186×*L_s_* m) or measured on the subject,
*l*_2_ length of the forearm (≈(0.146+0.108)×*L_s_* m) or measured on the subject,
*l_c_*_1_ length from shoulder to center of mass of the arm (≈*l*_1_×0.436 m),
*l_c_*_2_ length from shoulder to center of mass of the forearm (≈*l*_2_×0.682 m),
*g* gravity field (≈9.81 m.s^−2^),
*I*_1_ inertia of the arm w.r.t center of mass (≈*m*_1_×(*l*_1_×0.322)^2^ kg.m^2^),
*I*_2_ inertia of the forearm w.r.t center of mass (≈*m*_2_×(*l*_2_×0.468)^2^ kg.m^2^).

The variables will be denoted as follows: 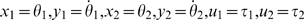.

Let *H* and *B* denote the matrices:
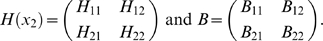



Then, the control system can be rewritten as:
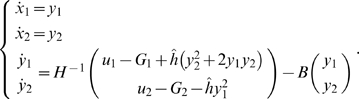



For all *x*_2_ *H* is invertible. We set
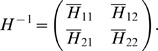



The explicit expression of the elements of *H* ^−1^ is:
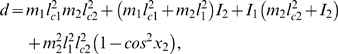
and:
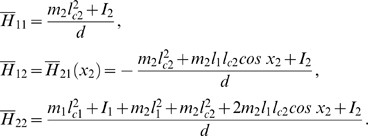



For both Examples 1 and 2 it will be interesting to consider the “small angles assumption”, i.e., the linearization of the system around some reference angles and zero velocity.

Since in the paper we only consider pointing movements, i.e., going (in short time *T*) from some initial condition (*x*, *x* ˙) = (*x_s_*, 0) to some terminal condition (*x_t_*,0) (both equilibria of the system), this assumption corresponds to the fact that *x_t_* is close to *x_s_*.

With this assumption, both examples become much simpler, as expressed by Equations 14 and 15 below, and calculations can be done explicitly. Without it, some numerical steps remain. Nevertheless in these numerical steps it is of great interest to know a priori the qualitative scenario for the optimal controls, which is of course the same as with the small angles assumption. Thus, although the small angles assumption may be irrelevant from an experimental point of view, it is useful for finding the optimal solution of the complete systems given in Examples 1 and 2.

***Example 3. One-degree of freedom, small angles assumption.*** Assuming the arm to be horizontal at the initial condition, we get cos(*x_s_*) = 1 and the linearized system around (*x_s_*,0) is the following standard linear control system:
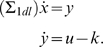
(14)


***Example 4. Two-degree of freedom, small angles assumption.*** As in the previous example, we neglect friction terms. Therefore, in the linearization around an equilibrium point (*x*, *x* ˙) = (*x*, *y*) = (*x_s_*, 0), we get no occurrence of *y*: the linear part is zero and the quadratic part in *y* disappears at *y* = 0. Therefore, the linearized system is of the following form:
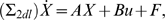
(15)where *X* = (*x*,*y*) and *A*, *B*, *F* are of the form:
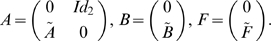



Here, 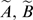 are 2×2 matrices, 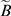 is invertible and 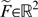. It follows that (Σ_2*dl*_) is a controllable linear system. Note also that the original system (Σ_2*d*_) is feedback-linearizable. This last point is important at several places in the paper.

#### Mathematical prerequisites

Our theory of inactivation relies on three mathematical facts:

- Thom's transversality theory,
- The classical Pontryagin's Maximum Principle,
- The characterization of the extrema of non-smooth (but Lipschitz-continuous) functions.

For the sake of completeness, we restate here the main points 2 and 3. Well-written introductions to Transversality theory may be found in [40], [58], and [59].

***Extrema of a strictly-convex (locally) Lipschitz-continuous function.*** Let *f*(*u*) be a locally Lipschitz function of the variable 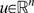. It means that, in restriction to any compact set Ω of 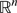:
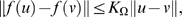
for a certain constant *K*_Ω_ depending on Ω. Here, we use any arbitrary norm over 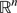. A locally Lipschitz function is clearly continuous. It is a less obvious fact that it is also almost everywhere differentiable.

Following F. Clarke [42], we define the generalized gradient of *f* at *u*_0_ denoted by ∂*_u_f*(*u*_0_), as the convex envelop of all possible limits of derivatives of *f* at points 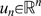, and *u_n_*→*u*_0_. Note that, in general, ∂*_u_f*(*u*_0_) is a set. Of course, if *f* is continuously differentiable on a neighborhood of *u*_0_, its generalized gradient at *u*_0_ coincides with the usual one and the set is reduced to a singleton.

For instance, if 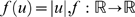, then *f* is everywhere continuously differentiable except at *u* = 0, and possible values for the derivative are ±1. Then the generalized gradient ∂*_u_f* is:
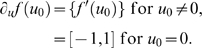



The important facts for us will be the following:

(F1) In restriction to an arbitrary compact subset, a strictly convex function has a minimum which is attained uniquely;
(F2) A necessary and sufficient condition for u* to be the point where *f* reaches its (absolute) minimum is:
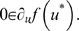
(16)

Note that, at a point where *f* is continuously differentiable in the classical sense, this condition is equivalent to the classical one: the gradient must be zero.

***Pontryagin's and Clarke's Maximum principle.*** The Maximum Principle gives necessary conditions of optimality for optimal control problems. For our problem 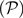, when Φ(*X*,*u*) and *ϕ*(*A* ˙*w*, *X*, *u*) are smooth w.r.t. *X*, we can use the original Pontryagin's version whose statement is as follows.

Denote by
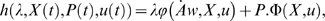
the Hamiltonian of the problem, where *λ*≤0.

If (*X*(*t*),u*(*t*)) is an optimal trajectory of the problem, then there exists 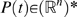 (dual space of 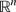), *P*(*t*) being absolutely continuous, (*λ*,*P*(*t*)) never vanishing, such that:

1. optimal trajectories meet the Hamiltonian equations:
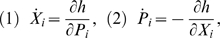
(17)
2. the Hamiltonian *h*(*λ*,*X*(*t*),*P*(*t*),u*(*t*)) reaches its maximum with respect to *u* at each time *t* ∈ [0,*T*].

Note that (*λ*,*P*(*t*)) is called the adjoint vector and that, in fixed time *T*, the Hamiltonian *h* does not necessarily vanish.

When moreover Φ(*X*,*u*) or *ϕ*(*A* ˙*w*, *X*, *u*) is non-smooth with respect to *X* (but at least Lipschitz continuous), which will happen in the section where we consider the case of gradient constraints on the control, the adjoint Equation 17 (2), has to be replaced by its non-smooth version (see [42]):
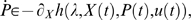
where ∂*_X_h* denotes Clarke's generalized gradient of *h* with respect to *X*.

Also, even in the classical case, since we assume the cost function *ϕ*(*A* ˙*w*(*u*), *X*, *u*) to be strictly convex w.r.t. *u*, the condition of maximizing the Hamiltonian *h* w.r.t. *u* can be replaced by (if the maximum is not attained on the constraints):
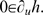
(18)


In any case, even if the cost function is not strictly convex w.r.t. *u*, this condition is necessary in order to maximize the Hamiltonian.

***Nonexistence of abnormal trajectories.*** In this section we consider a general exactly-fully-actuated system. An *extremal* is a trajectory of the system meeting the necessary conditions provided by the Maximum Principle. A *singular* extremal is an extremal corresponding to *λ* = 0 (or equivalently, to the minimum-time problem). Extremals corresponding to *λ*<0 are called *regular*.

A *bang* extremal is an extremal such that for almost all *t* ∈ [0,*T*], one of the control variables *u_i_* can take the two values 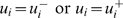 only.

Here, an *abnormal* extremal is a singular extremal which is not bang.

Since our system is feedback-linearizable, it admits no such abnormal extremal. To the best of our knowledge, this fact has been noticed for the first time in [60]. Let us briefly recall its proof.

Setting *x* ˙ = *y*, *P* = (*p*,*q*) and *X* = (*x*,*y*), our Hamiltonian *h*, with *λ* = 0, can be rewritten as:
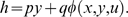
(19)


Note also that, for our mechanical systems, *ϕ* is linear with respect to *u*, and *u* enters via the term *M*(*x*)^−1^ (Equation 5).

Therefore the condition of maximum of the Hamiltonian for an abnormal extremal gives *q* = 0. This has to be true along the abnormal trajectory (not pointwise): *q*(*t*) = 0 for all *t*. Therefore, differentiating, we get that *q* ˙(*t*) = 0 also, but by the Hamiltonian equations:
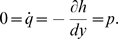



Then, *p*(*t*) has also to be zero. This is a contradiction with the maximum principle, which prescribes that (*λ*,*p*(*t*),*q*(*t*)) never vanishes.

#### The statement of the Inactivation Principle

A rough statement of the Inactivation Principle is as follows: provided that the total duration *T* of the motion is not too large (compared to the minimum time *T*_min_), then there is partial inactivation along an optimal trajectory minimizing a compromise *J*(*u*) between the absolute work and a comfort term (*J*(*u*) of the form given by Equation 7, or more generally Equation 8). Moreover, simultaneous periods of inactivation of all controls may appear in a stable way (stable w.r.t small smooth perturbations of the cost, or of the system).

Note that *T*_min_ is the minimum time to reach the target from the source. It does exist and it is reached by a bang-bang control, due to absolute bounds on the values of controls.

This is not a theorem, but a principle. To transform the statement into a theorem, we need precise technical assumptions.

Let us consider some optimal trajectory (*X*,u*) defined on [0,*T*], and meeting the following two technical assumptions (*H*_1_,*H*_2_):

(*H*_1_) Continuity of optimal control: u*(*t*) is continuous on [0,*T*],
(*H*_2_) Change of sign for optimal control: some component 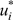 of optimal control changes sign at some time *t_c_* ∈ ]0,*T*[,while *y_i_* (*t*) keeps constant sign. It means that there are some times *t*_1_ *t*_2_, *t*_1_<*t_c_*<*t*_2_, such that 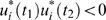 and *y_i_*(*t*)≠0 for *t*_1_≤*t*≤*t*_2_.

#### Theorem 2. (Inactivation Principle)

*Along a regular optimal trajectory of* 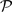 *meeting hypotheses* (*H*_1_,*H*_2_) *there is partial inactivation. If all regular extremals are continuous, then some of them passing through an arbitrary* 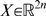 *have total inactivation.*

*Proof.* Along the optimal trajectory, the Hamiltonian *h* of the optimal control problem has to be maximum, which means by Equation 18 that 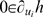 for all *i* = 1,…,*p*. But,
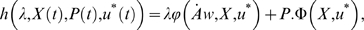
and *λ*<0 since we consider regular trajectories only. The maximum condition for the Hamiltonian gives:
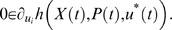
(20)


The variables *X*(*t*) and *P*(*t*) being also continuous, the quantity 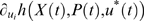 is an interval *I*(*t*) (degenerating to a point as soon as 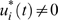 and moving continuously with the time *t*. At a time *t_c_* when 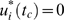, it is a nontrivial time interval *I*(*t_c_*), since 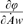 and *λ* are both different from zero. Hence, since 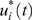 changes sign at *t_c_*, it takes a certain strictly positive amount of time to cross *I*(*t_c_*). Then 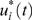 remains exactly equal to zero during some nontrivial time interval. This is partial inactivation. Continuing, we take an arbitrary *X* = (*x*,*y*), with *y_i_*≠0 for all *i* = 1,…,*n* and *λ* = −1. We denote by (*M*(*x*)^−1^)*_i_* the *i*th column of the invertible matrix *M*(*x*)^−1^. Then, for *u* = 0, we compute the set 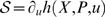. If we set *P* = (*p*,*q*), then due to the fact that 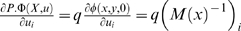, we can choose *q* in order that 0 be exactly the center of the set 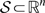, which is a hypercube with nonempty interior. It is clear by construction that the extremals starting from this point (*X*,*P*,0), if continuous, have total inactivation.

**Figure 9:**
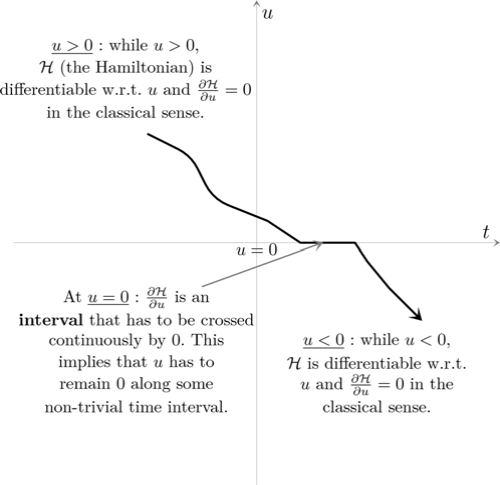
Intuitive illustration of the Inactivation Principle proof.

This proof is illustrated in Figure 9.

Let us examine now the validity of the assumptions (*H*_1_,*H*_2_) above. We have first the following result.

#### Lemma 1

The optimal controls u*(*t*) *corresponding to regular trajectories of* 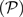 *are continuous w.r.t. t.*

***Proof.*** The lemma 11 in [61] states the following. Consider a function *f*: 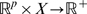, where *X* is a manifold and *f* is continuous, with the additional property that for each compact *K* ⊂ *X*, the restriction 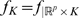 is proper. Then, 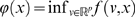 is a well defined mapping, continuous over *X*. Examination of the proof of this result shows that it holds also for *f*: 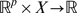. We apply this lemma to our Hamiltonian *h*. Due to assumption A and to the fact that *λ*<0, *h*(*t*,*u*) = *h*(*X*(*t*),*P*(*t*),*u*) is a strictly concave function of *u*. Moreover, it is continuous since *X*(*t*) and *P*(*t*) are continuous functions of *t*. Let *u*(*t*_0_) be a discontinuity value of the optimal control *u*(*t*). It means that we can find a sequence *t_n_*→*t*_0_ such that *u*(*t_n_*)→*u* ^≠*u*(*t*_0_). Applying the above-mentioned lemma to −*h*(*t*,*u*), where *u* here is the variable *ν* in the lemma, we get that *t*→−*h*(*t*,*u*(*t*)) is a continuous map. But the minimum being unique, this contradicts the assumption *u*(*t_n_*)→*u* ^≠*u*(*t*_0_).

Note that in general, optimal control may not be continuous: consider Example 1 with *T* = *T*_min_ (the minimum time). Since there is no abnormal trajectory, the optimal control (which is also the minimum-time control) jumps between the bounds *u* ^−^ and *u* ^+^.

This means that assumption (*H*_1_) holds provided that the optimal trajectory is regular, which is the case in general when *T*>*T*_min_. This is verifiable for instance for a cost of type compromise *Aw*/*Ae*.

Indeed, consider a singular extremal with *T*>*T*_min_. Then this extremal corresponds to an extremal of the minimum-time problem. Thus, 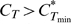, where *C_T_* and 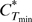 are the cost of the singular extremal and the optimal cost of the minimum-time problem, respectively. Since the value of minimum cost is a strictly decreasing function of *T* on the time interval [*T*_min_,+∞[ (see Theorem 1 in [43]), there is a contradiction.

Assumption (*H*_2_) (the change of sign of the optimal control) is also true in general. This can be proved in the following way.

The input-state mapping
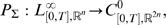
is continuous for the *-weak topology over 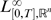 [59]. When *T*→*T*_min_ from above, we consider the restriction *u_T_* of the optimal control to the interval [0,*T*_min_]. This defines a sequence of controls that (by boundedness) we can assume to be *-weak convergent to some control u*(*t*). By construction, this u*(*t*) is a minimum time control. Since *u_T_*(*t*) is continuous, if *T* is close enough to *T*_min_, *u_T_*(*t*) has sign changes close to the sign changes of the minimum-time control u*(*t*).

The fact that the minimum time control u*(*t*) has changes of sign can be checked directly.

For instance, in Example 1, minimum time control can only commute between the values *u* ^−^,*u* ^+^. These values are large enough. Hence if there is no commutation, the control is constant and large. Therefore *y* ˙(*t*) has constant sign and *y*(*t*) cannot go from zero to zero.

*Remark 4.* The previous reasoning shows that in general inactivation is located around instants that are close to the instants where the minimum time-control changes sign (commutes). This reasoning also shows that inactivation occurs automatically for a duration *T* of the motion sufficiently close to the minimum time *T*_min_. This is coherent with practical observations showing that for larger *T*, simultaneous inactivation of agonistic and antagonistic muscles disappear.

#### The necessity of the absolute work term for inactivation

The purpose of this section is to show that, for the occurrence of inactivation in optimal trajectories, it is necessary that the minimized integral cost contains a term “like the absolute work”. This means a term with some non-smoothness at *u* = 0 (remind that *u_i_* = 0 corresponds to inactivation at the level of the *i*th degree of freedom).

We fix a “source-point” 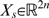, a “target-point” 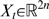, and a time *T*>0. The points *X_s_* and *X_t_* correspond to zero velocity, i.e., are of the form (*x*, *x* ˙) with *x* ˙ = 0. Given a function *f* on 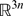, we define the following optimal control problem:
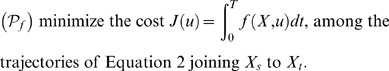



We also set *F*(*X*) = Φ(*X*,0).

#### Theorem 3

*There exists an open and dense subset O of* 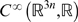 *(endowed with the C^∞^ Whitney topology) such that, if f* ∈ *O, then* 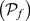 *does not admit minimizing controls which vanish on a subinterval of* [0,*T*], *except maybe if the associated trajectory is an equilibrium point of F. In addition, for every integer m, the set O can be chosen so that its complement has codimension larger than m.*

*Remark 5.* (1) In the previous theorem, we use the Whitney topology over the set of cost functions *f* to be minimized. It is the usual topology in this setting. If we restrict to a fixed compact set, it is equivalent to consider the usual topology of *C* ^∞^ convergence over this compact set. (2) The fact that the bad set (the set of exceptional cost functions for which inactivation can be optimal) has codimension infinity (i.e., codimension larger than *m*, for all *m*) means that the good set is extremely large.

The proof of this theorem is given in Supporting Information (Text S1).

The gist of the proof is the following: we assume that the cost function is smooth, and we show that (up to exceptional and unstable cases for the cost), the only optimal trajectories that are constant can be either equilibria trajectories or bang trajectories (i.e., trajectories lying in the boundary of the control set). This is done by using transversality arguments: Thom's transversality theorem simply states in precise mathematical terms that, “generically”, mathematical objects are in “general position”. For instance (see Page 67 in [40]), consider the following statement: if *f* is a continuously differentiable function, “almost all” horizontal lines are nowhere tangent to the graph of *f*. This statement illustrates a type of reasoning that is common in differential topology. Transversality gives the necessary framework to justify such kinds of properties.

Roughly speaking, for inactivation to be optimal in a stable way (i.e., remain optimal while not overly perturbing the cost to be minimized) then it is necessary that the cost function *f* is non-smooth at *u* = 0.

A similar theorem holds also for partial inactivation (inactivation of one control at least, on some nontrivial time-interval). But in that case, for technical reasons, we have to restrict to the open set 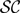 of *C* ^∞^-smooth functions all *f* that are moreover strictly convex with respect to *u*. Here and only here, by strictly convex, we mean the assumption that the Hessian of *f* w.r.t. *u* is everywhere positive-definite. This assumption clearly defines an open subset 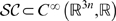 for the Whitney topology.

#### Theorem 4

*There exists an open and dense subset O′ of* 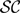 *such that, if f* ∈ *O′, then* 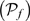 *does not admit minimizing controls, a component of which vanishes on a subinterval of* [0,*T*] *(again except maybe if the associated trajectory is an equilibrium point of F).*

The proof of this more difficult result is also given in Supporting Information (Text S1).

#### Detailed results for the one and two-degree of freedom arms

***The 1-dof case, n = 1.*** This case has been extensively studied in [43]. Here we just revisit the main results. Notice that the following results are obtained with Example 3 by minimizing the compromise *Aw*/*Ae*.

In Figure 1, we have depicted the results we get for an upward motion in the case of gradient constraints on the control. This is the reason why we have moreover a gradient constraint reached at the beginning and at the end of the motion. However, in this figure, one can see very clearly the inactivation interval which illustrates the Inactivation Principle.

We obtained the following seven different optimal strategies for an upward movement, the equations of which are established from Pontryagin's Maximum Principle. Each of them is an optimal solution of the problem, depending on the explicit values of the parameters, like the movement duration *T* or the weighting parameter *α*.

In the following, (*p*,*q*) will denote the adjoint vector, and (*p*_0_,*q*_0_) will denote its initial value at *t* = 0.

The 7 qualitative types of optimal strategies are denoted by *S_j_*, *j* = 1,…,7 and correspond to the following sequences of controls:

(*S*_1_) (bang-max, bang-min):
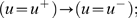

(*S*_2_) The most general strategy (regular-bang, regular non-bang, inactive, regular non-bang, regular-bang):
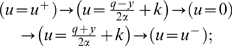

(*S*_3_) (regular non-bang, inactive, regular non-bang, regular-bang):
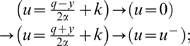

(*S*_4_) (regular-bang, regular non-bang, inactive, regular non-bang):
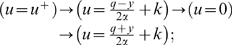

(*S*_5_) (regular non-bang, inactive, regular non-bang):
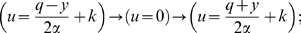

(*S*_6_) (regular-bang, regular non-bang):
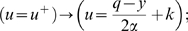

(*S*_7_) (regular non-bang only):
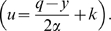

The “inactive” pieces are inactivation periods, *u* = 0.

In the following we describe in details the strategies (*S*_1_) (minimum time) and (*S*_2_).

We will use the notations *u_i_*(*t*), *q_i_*(*t*), *x_i_*(t), *y_i_*(*t*), for *t* ∈ [0,*τ_i_*] and *i*≥1 for the functions *u*,*q*,*x*,*y* on the interval 
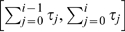
 with *τ*_0_ = 0. For instance, *u*_2_(*t*) means *u*(*t*+*τ*_1_) for *t* ∈ [0,*τ*_2_] and *u*_3_(*t*) means *u*(*t*+*τ*_1_+*τ*_2_) for *t* ∈ [0,*τ*_3_].

#### Case S_1_. Fastest possible movements, critical time *T_c_* = *T*_min_

This is the singular case, corresponding to the quickest possible movement. This solution is bang, i.e., depends only upon the constraints *u* ^+^, *u* ^−^.

The corresponding equations for the solutions are the following, assuming the small angles approximation:

- For *t* ∈ [0,*τ*_1_]
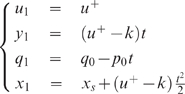

- For *t* ∈ [0,*T_c_*−*τ*_1_] (*τ*_2_ = *T_c_*−*τ*_1_)
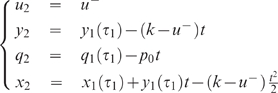
with
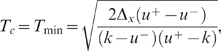
and commutation time *τ*_1_,
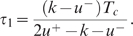

#### Case S_2_. The most general strategy, five-piece trajectories

this case is also the most complicated scenario and it appears when movement duration is close to *T*_min_, but with *T*>*T*_min_.

- For *t* ∈ [0,*τ*_1_]
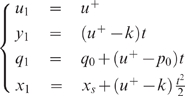

- For *t* ∈ [0,*τ*_2_]
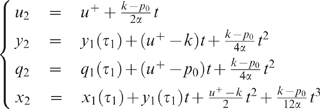

- For *t* ∈ [0,*τ*_3_]
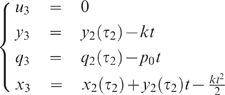

- For *t* ∈ [0,*τ*_4_]
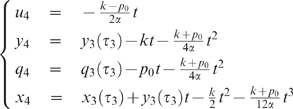

- For *t* ∈ [0,*τ*_5_]
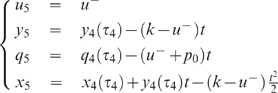

The commutation times *τ_i_* meet:
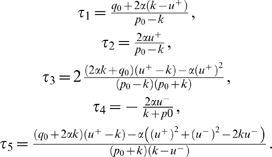



Of course, we have *τ_i_*>0 for all *i* and 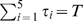. This implies several constraints on *p*_0_ and *q*_0_. The initial adjoint vector can be computed by requiring that *y*_5_(*τ*_5_) = 0 and *x*_5_(*τ*_5_) = *x_t_*. Explicit formulae for *p*_0_ and *q*_0_ cannot be obtained but it is numerically easy to compute these values, and to check if they are compatible with the conditions above.

**Figure 10:**
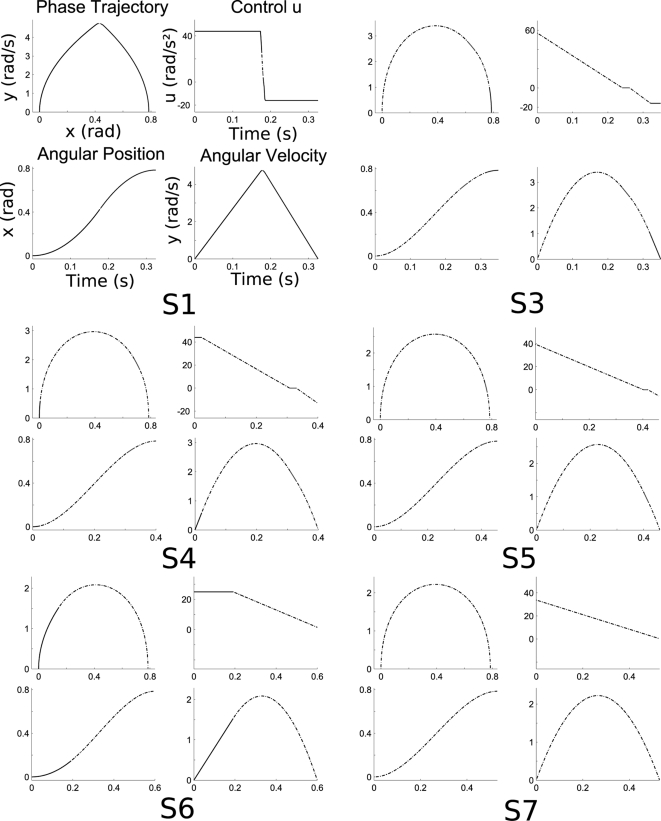
Different optimal strategies in the 1-dof case, depending on the movement duration *T*. The strategy *S*1 depicts the fastest movement w.r.t. the bounds imposed on the control. Strategy *S*2 was depicted in Figure 1 with gradient constraints on the control *u*. Strategies *S*3, *S*4, and *S*5 show inactivation phases (as well as *S*2). An inactivation phase corresponds to the period where the control signal *u* is zero. When *T* becomes large (*T*≥0.6 s in this case), the inactivation disappears (*S*6 and *S*7 strategies) according to experimental findings. The angular position and velocity and the control signal are given in radians, rad/s, and rad/s^2^, respectively. Note that the control signal *u* corresponds to the ratio between the net torque acting at shoulder joint and the arm's moment of inertia.

Figure 10 illustrates the different strategies, except the most general, strategy (*S*_2_), which was depicted in Figure 1 in the case of gradient constraints on the control.

As shown in this figure, inactivation occurs for *T* not too large. The time *T*_2_ at which total inactivation disappears may be of importance for experimenters.

We have computed it using the small angles assumption:
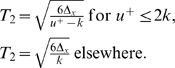



In this analysis, computations are tedious, but quite easy: optimal control of a linear system with strictly convex (piecewise quadratic) cost function. Hence all the results in this section are obtained directly with the Maximum Principle.

Importantly, it can be shown (by comparisons) that the whole optimal trajectories are entirely in {*y*≥0} or {*y*≤0}. Therefore, there is just non-smoothness w.r.t. *u*, and we need only the usual Pontryagin's Maximum Principle (no necessity of Clarke's version in this case).

Let us give more insights concerning the optimal synthesis. Consider the Hamiltonian H of the problem:
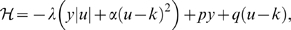
(21)where *λ*≥0 is the constant additional adjoint variable, and (*p*,*q*) is the adjoint vector to (*x*,*y*). We can take *λ* = 1 since singular extremals do not appear for *T*>*T*_min_.

We set *z* = *q*−*y* and *w* = *q*+*y*. The condition *y*≥0 is now *w*≥*z*.

**Figure 11:**
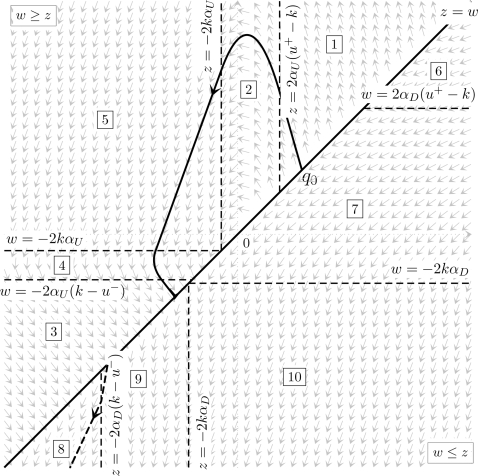
Phase portrait for *p*_0_≥*k* in the plane (*z*,*w*). The bisector (*z* = *w*) corresponds to the set of velocities equal to zero. The upper and lower semi-plane corresponds to positive and negative angular velocities, respectively. An optimal path starts and ends on this line. This figure illustrates the optimal phase portrait corresponding to the *S*2 strategy (for an upward motion). Regions are denoted by boxed numbers and the commutation times correspond to switches between regions. For instance Region 5 corresponds to the inactivation region (i.e., the control signal is zero here). Note that the different strategies illustrated in Figure 10 are easily understood with this phase portrait, since optimal paths may start and end in different regions. The constants *k*, *α_U_*, *α_D_*, and *u* ^+^ and *u* ^−^ are parameters depending respectively on the mechanical model of the arm, the coefficients involved in our cost function, and the boundary values imposed on the control *u*.

Figure 11 shows the (*z*,*w*) phase portrait of the optimal trajectories obtained from the maximization of the Hamiltonian w.r.t. *u* when *p*_0_>*k*. The typical trajectory drawn in the half-plane *y*≥0 (i.e., *w*≥*z*) corresponds to the most general trajectories (*S*_2_).

***The 2-dof case.*** Again, we want to minimize the compromise *Aw*/*Ae*.

We write the Hamiltonian in the 2-dof case, omitting dependence of different terms w.r.t. variables *x*_1_, *x*_2_. The adjoint vector is denoted here (*p*_1_,*p*_2_,*q*_1_,*q*_2_).
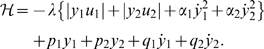



Then Pontryagin's equations of the Maximum Principle are:
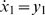


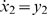


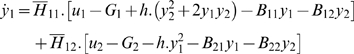


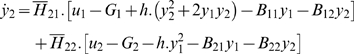


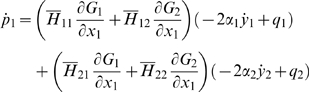


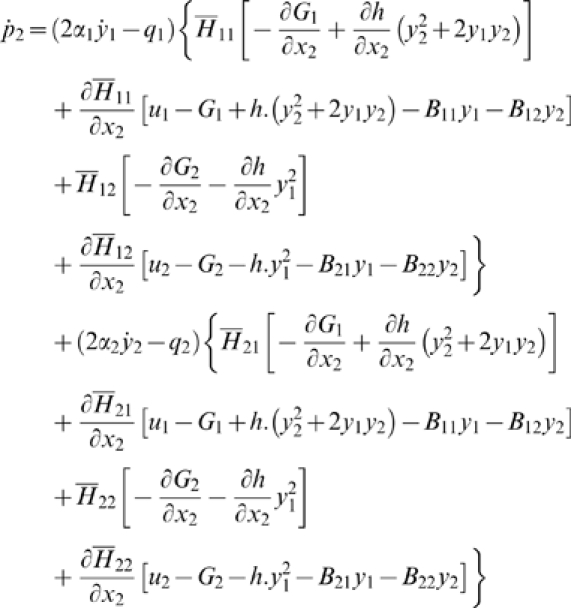


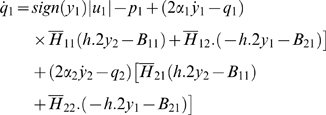


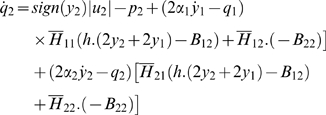



Singular extremals (*λ* = 0) again do not appear for *T*>*T*_min_. Hence we take *λ* = 1 and we have to maximize the following w.r.t. *u*_1_,*u*_2_:
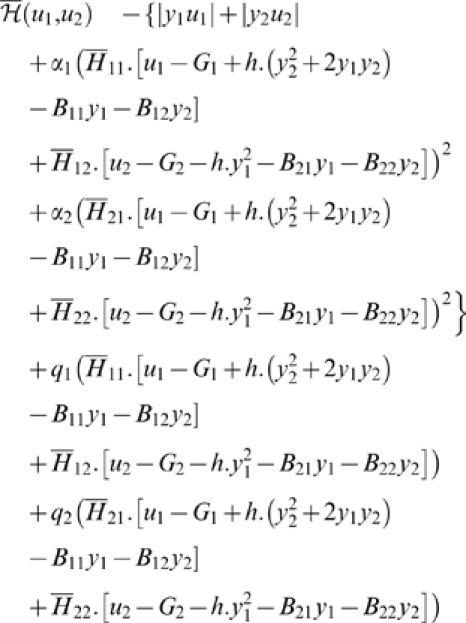



We discuss this maximization in terms of the 9 regions in the *u*_1_,*u*_2_ plane corresponding to the “stratification by the sign of coordinates”.

This is done in Supporting Information (Text S1) where we explain how to compute the extremals.

Notice that, as in the 1-dof case, many different strategies can occur, with or without inactivation at each joint. The case of total inactivation of both controls is also possible.

Numerical solutions are depicted in Figure 2.

#### The case of gradient constraints on the torques

This is a rather simple extension of the theory. The results obtained in the 1-dof case have already been depicted in Figure 1. Here, we explain what happens in this case only, however the case of 2-dof is similar.

In this problem, we require moreover that the derivative of the torque *u* is bounded.

We introduce the new control *v* = *u* ˙ and the problem may be rewritten, as (taking possibly frictions into account):
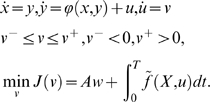



Now the cost function is not differentiable anymore with respect to the state (in place of the control in previous sections). Therefore, Clarke's non-smooth version of the Maximum Principle is needed [42].

If (*p*,*q*,*r*) denotes now the adjoint variables, we get:
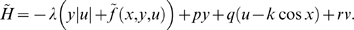
Once again, *x*,*y*,*p*,*q*,*r*,*u* are continuous (by nature now, just as classical solutions of differential equations). The *a priori* fact that *y* remains positive is just checked numerically. However, it is expectable from the results obtained without gradient constraints on the torques.

Also, for similar reasons as in a previous subsection, the abnormal extremals may be excluded: maximality of the Hamiltonian for non-bang trajectories implies that *r* is identically zero, which implies, with two successive differentiations, that *q* and *p* respectively are also identically zero. Total adjoint vector is zero, which contradicts the maximum principle. Hence we may assume *λ* = 1.

We assume that the gradient constraints *ν* ^−^ and *ν* ^+^ are large enough for the optimal control to be of the following type: gradient constraints which are active only at the beginning and at the end of the motion. If we refer to the scenario occurring in 1-dof case, this should be what happens: without the gradient constraints, the gradient is never large. Then, there will be saturation of the gradient constraints only because of the jumps at the beginning and at the end of the motion. Numerical computations confirm this scenario, as illustrated in Figure 1.

For instance, consider that *x_t_*>*x_s_*, i.e., an upward movement. Then, to connect (in optimal way) the source (*x_s_*,0,*u_s_*) to the target (*x_t_*,0,*u_t_*), where *u_s_* and *u_t_* are the stationary torques corresponding respectively to the equilibrium positions *x_s_* and *x_t_*, the strategy must be as follows: *ν* = *ν* ^+^, for 0≤*t*<*T*_1_; *ν* ^−^<*ν*<*ν* ^+^ for *T*_1_≤*t*≤*T*_2_; *ν* = *ν* ^−^ for *T*_2_<*t*≤*T*.

Therefore, inside the interval [*T*_1_,*T*_2_], the Hamiltonian is maximum w.r.t. *ν* and we must have *r*(*t*) = 0. Therefore 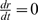. But by Clarke's maximum principle, it means that 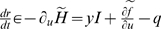, in which *I* is the Clarke's gradient of the absolute value function at zero, i.e., *I* = [−1,1].

Since 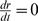, we conclude:
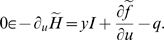
This equation was exactly the cause of the presence of inactivation when we proved the Inactivation Principle: it is Equation 20.

Therefore, the inactivation phenomenon persists under torque gradient constraints.

Notice that, adding gradient constraints also permits getting smoother velocity profiles with zero-acceleration at the initial and final times.

#### The Inactivation Principle for agonistic-antagonistic torques

The purpose here is to show that the Inactivation Principle persists when considering that two opposing torques act at each joint (one agonistic and one antagonistic). This is the case *m* = 2*n* of the Theoretical Analysis Subsection.

For this analysis, we consider that *u* = *u*_1_−*u*_2_, where 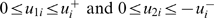. Then *u*_1*i*_ (resp. *u*_2*i*_) are the agonistic (resp. antagonistic) generalized torque applied at the *i*th degree of freedom.

For the case of net torque *u*, the cost that we consider is the compromise given by Equation 4, i.e.,
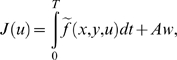
with:
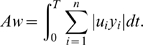



It means that, for agonistic-antagonistic torques, we shall minimize:
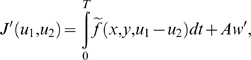
where *Aw*′ is the total absolute work of external torques:
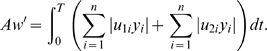



Firstly, let us assume that *u*_1_, *u*_2_ minimize *J*′, with optimal value *J*′*. Consider *u* = *u*_1_−*u*_2_.

Clearly, *u* applied to the system:
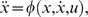
(22)and, *u*_1_, *u*_2_ applied to the system:
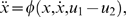
(23)produce identical *x*-trajectories.

Therefore,
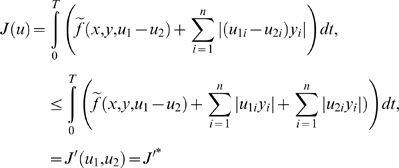
This shows that the minimum J* = min*_u_J*(*u*)≤*J*′*.

Conversely, assume that *u* attains the minimum J* of *J*(*u*). We define *u*_1_, *u*_2_ from *u* as follows (for *i* = 1..*n*):
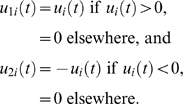
(24)


Again *u*_1_−*u*_2_ = *u*. Hence applying *u* to Equation 22 produces the same *x*-trajectory as applying *u*_1_−*u*_2_ to Equation 23. Therefore, by definition of *u*_1_, *u*_2_, we have:
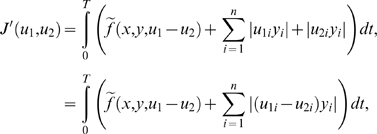



It means that:
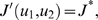
(25)which implies that *J*′*≤J*. It is now clear that *J*′* = J*, and also by Equation 25 the minimum is reached by in Equation 24.

Notably, by construction, the torques *u*_1*i*_, *u*_2*i*_ have simultaneous inactivation only when *u_i_* = 0, for *i* = 1..*n*.

We have proved the following theorem:

#### Theorem 5. (Simultaneous inactivation for agonistic-antagonistic torques)

*In the case of agonistic-antagonistic torques, minimizing a cost containing the absolute work leads to a simultaneous inactivation of both torques, exactly at the same times where the optimal net torque is inactive.*

#### Dynamics of the muscles and the triphasic pattern

In this section, we still consider agonistic-antagonistic torques, but we assume some dynamics on each muscle. For the sake of simplicity, we assume a first order dynamics on the muscles, but this restriction is not crucial. Also, we present the results in the 1-dof case (*n* = 1) and we make the small angles assumption, in order to make the computations more tractable.

As in previous subsections, we minimize the compromise *Aw*/*Ae*.

Then, adding the first order time constants *σ*_1_, *σ*_2_ on both muscles, we get the following control system:
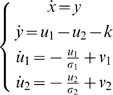
(26)with *ν*_1_, *ν*_2_≥0.

We look for the minimum 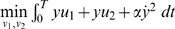.

For this, we use the a priori fact (which is checked numerically) that, as in the case of torque control, *y* remains positive during the upward motion [43]. The Hamiltonian may be written as:
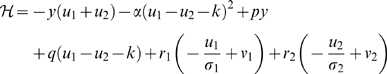



At this point, there is an important technical detail that physiologically makes sense. It can be understood as muscular co-activation at the end of the motion, a well know phenomenon in physiology.

Due to the first order linear dynamics on the muscles, and the constraints *u_i_*≥0, we can only go back to zero asymptotically. Therefore, the terminal condition 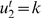 is impossible, i.e., the antagonistic torque cannot go back to exactly zero at the end of the movement.

Hence we require, with *ε*>0:
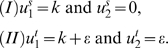
(27)


Notice that when modeling muscles dynamics, the initial and final values of both agonistic and antagonistic torques must be specified in order to maintain the arm at equilibrium.

**Figure 12:**
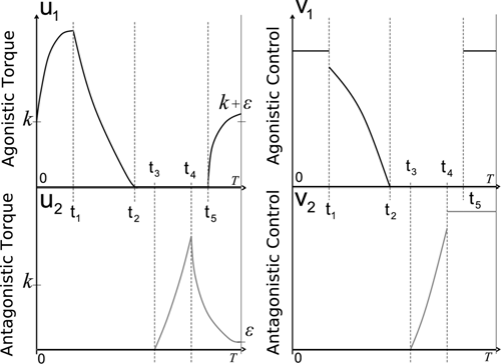
Optimal Triphasic Pattern. Illustration of the optimal behavior of a 1-dof arm, under the small angles assumption and with a pair of agonistic and antagonistic muscles, modeled by first-order dynamics. The subscripts 1 and 2 denote the flexor and extensor muscles, respectively. The triphasic pattern is an agonistic burst, followed by an antagonistic burst, and again an agonistic burst. The inactivation occurs between the first agonistic and antagonistic bursts. The times *t_i_* denote the commutation times. The left graphs illustrates the behavior of the angular torques (*u*). The right graphs illustrate the behavior of the control signals (*ν*), that are the input signals for muscles contractions (i.e., the signals driven by motoneurons). All signals are plotted with respect to time *t* varying between 0 and *T*.

Requirement (*II*) is the co-activation at terminal time *T*. Then, explicit computations with the Maximum Principle, together with a numerical research of the commutation times, show that the optimal scenario is as shown in Figure 12.

One can recognize the classical scenario called “triphasic pattern” [62], namely: an agonistic burst followed by an antagonistic burst followed again by an agonistic burst (the scenario ends with the above mentioned co-contraction of the muscles).

In fact, our theory shows that it may be called “quadriphasic pattern” since there is an inactivation interval between the first agonistic pulse and the antagonistic one.

## Supporting Information

Text S1 Some Mathematical Details and Technical Proofs
(0.12 MB PDF)
