# Supplementary material for: The Inactivation Principle: Mathematical Solutions Minimizing the Absolute Work and Biological Implications for the Planning of Arm Movements
Source: PLoS Comput Biol. 2008 Oct 24;4(10):e1000194. doi: 10.1371/journal.pcbi.1000194 (PMC2561290; doi:10.1371/journal.pcbi.1000194)
Supplement: Text S1 — Some Mathematical Details and Technical Proofs (0.12 MB PDF) [file pcbi.1000194.s001.pdf]

Supporting Information (Text S1)

Some Mathematical Details and Technical Proofs

Bastien Berret, Christian Darlot, Frédéric Jean, Thierry Pozzo, Charalambos Papaxanthis, and Jean Paul Gauthier

Proof of Theorem 4

The proof is based upon Thom's transversality theorem. We will then make the computations in the spaces of jets. For a positive integer m and a pair $(X, u) \in \mathbb{R}^{2n} \times \mathbb{R}^n$, we denote by $\mathcal{J}^m_{(X,u)}$ the space of m-jets at (X, u) of functions in $C^\infty(\mathbb{R}^{3n}, \mathbb{R})$.

Fix now a point $X^0 \in \mathbb{R}^{2n}$ which is not an equilibrium of the vector field F. We define $\mathcal{A}^m(X^0) \subset \mathcal{J}^m_{(X^0,0)}$ as the set of m-jets of functions $f \in C^\infty(\mathbb{R}^{3n}, \mathbb{R})$ such that the trajectory of Equation 11 issued from X^0 and associated to the control $u = 0$ is locally minimizing for the optimal control problem $(\mathcal{P}_f)$.

Lemma 1. *$\mathcal{A}^m(X^0)$ is contained in a vector subspace of $\mathcal{J}^m_{(X^0,0)}$ of codimension $n(m-2)$.*

Proof. Without lack of generality we assume $X^0 = 0$. Let $j^m_0 f$ be a m-jet in $\mathcal{A}^m(0)$. By definition of $\mathcal{A}^m(0)$, the trajectory $X(\cdot)$ of F issued from 0 minimizes the problem $(\mathcal{P}_f)$ on an interval $I = [0, s]$. Thus $X(\cdot)$ satisfies Pontryagin's Maximum Principle on I: there exists a smooth function $P = (p, q) : I \to \mathbb{R}^n \times \mathbb{R}^n$ (the smoothness of P results from that of X) and $\lambda \geq 0$ such that, for all $t \in I$, $(P(t), \lambda) \neq 0$ and:

(P1) $\quad \dot{P}(t)^T = -\frac{\partial H}{\partial X}(X(t), P(t), \lambda, 0),$

(P2) $\quad H(X(t), P(t), \lambda, 0) = \max_{v \in U} H(X(t), P(t), \lambda, v),$

where the Hamiltonian of the problem is:

$$H(X, P, \lambda, u) = p^T y + q^T \phi(X, u) - \lambda f(X, u).$$

Note that, since $0 \in int\, U$, property (P2) implies $\frac{\partial H}{\partial u}(X(t), P(t), \lambda, 0) = 0$. It follows:

$$q(t)^T = \lambda \frac{\partial f}{\partial u}(X(t), 0) \frac{\partial \phi}{\partial u}(X(t), 0)^{-1}.$$

If $\lambda = 0$, then $q \equiv 0$. From $\dot{q} \equiv 0$ and (P1) we deduce $p \equiv 0$ and then $(P, \lambda) \equiv 0$, which is impossible. Thus λ is positive and a standard argument of homogeneity allows normalizing it to $\lambda = 1$. Finally, from respectively (P1) and (P2), the following holds on the interval I:

$$\begin{aligned} \dot{p}^T &= -q^T \frac{\partial \phi}{\partial x}(X, 0) - \frac{\partial f}{\partial x}(X, 0), \\ \dot{q}^T &= -p^T - q^T \frac{\partial \phi}{\partial y}(X, 0) - \frac{\partial f}{\partial y}(X, 0) \end{aligned} \quad (1)$$

and,

$$q^T = \frac{\partial f}{\partial u}(X, 0) \frac{\partial \phi}{\partial u}(X, 0)^{-1}. \quad (2)$$

Now, recall that on I the dynamic is $\dot{X} = F(X)$. Since $X^0 = 0$ is not an equilibrium point of F, we assume, up to a local change of the coordinates $X = (X_1, \ldots, X_{2n})$ on $\mathbb{R}^{2n}$, that $F = \frac{\partial}{\partial X_1}$. Differentiating Equation 1 with respect to time leads to:

$$\begin{aligned} \ddot{q}^T &= -\dot{p}^T - \dot{q}^T \frac{\partial \phi}{\partial y} - q^T \frac{\partial}{\partial X_1} \frac{\partial \phi}{\partial y} - \frac{\partial}{\partial X_1} \frac{\partial f}{\partial y} \\ &= -q^T \frac{\partial \phi}{\partial x} - \frac{\partial f}{\partial x} - \dot{q}^T \frac{\partial \phi}{\partial y} - q^T \frac{\partial}{\partial X_1} \frac{\partial \phi}{\partial y} \\ &\quad - \frac{\partial}{\partial X_1} \frac{\partial f}{\partial y}, \end{aligned} \tag{3}$$

in which we omit the evaluation at $(X, 0)$.

On the other hand, we can also obtain $\dot{q}^T$ and $\ddot{q}^T$ by differentiation of Equation 2:

$$\begin{aligned} \dot{q}^T &= \frac{\partial}{\partial X_1} \frac{\partial f}{\partial u} \times \left(\frac{\partial \phi}{\partial u}\right)^{-1} + \frac{\partial f}{\partial u} \times \frac{\partial}{\partial X_1} \left(\frac{\partial \phi}{\partial u}\right)^{-1} \\ \ddot{q}^T &= \frac{\partial^2}{\partial X_1^2} \frac{\partial f}{\partial u} \times \left(\frac{\partial \phi}{\partial u}\right)^{-1} + 2 \frac{\partial}{\partial X_1} \frac{\partial f}{\partial u} \times \frac{\partial}{\partial X_1} \left(\frac{\partial \phi}{\partial u}\right)^{-1} \\ &\quad + \frac{\partial f}{\partial u} \times \frac{\partial^2}{\partial X_1^2} \left(\frac{\partial \phi}{\partial u}\right)^{-1}. \end{aligned}$$

Substituting these expressions and Equation 2 into Equation 3, we eliminate q^T, $\dot{q}^T$, and $\ddot{q}^T$ and we obtain:

$$\frac{\partial^2}{\partial X_1^2} \frac{\partial f}{\partial u} + R_X \left(\frac{\partial}{\partial X_1} \frac{\partial f}{\partial u}, \frac{\partial f}{\partial u}, \frac{\partial}{\partial X_1} \frac{\partial f}{\partial X_i}, \frac{\partial f}{\partial X_i} \right) = 0 \qquad \text{on } I,$$

where, for every X, R_X is a linear mapping and $X \mapsto R_X$ is smooth. Successive derivations and evaluation of the derivatives at $t = 0$ (recall that $X(0) = 0$) lead to a system of equations of the form:

$$\begin{aligned} &\frac{\partial^k}{\partial X_1^k} \frac{\partial f}{\partial u}(0) + R^k \Big(\frac{\partial^j}{\partial X_1^j} \frac{\partial f}{\partial u}(0), \\ &\qquad \frac{\partial^j}{\partial X_1^j} \frac{\partial f}{\partial X_i}(0); j < k, 1 \leq i \leq 2n \Big) = 0, \quad k \geq 2, \end{aligned}$$

where each R^k is a linear mapping.

Thus we have proved $\mathcal{A}^m(0) \subset \ker \psi$, where $\psi : \mathcal{J}_0^m \to \mathbb{R}^{n(m-2)}$ is the linear mapping which associates

$$\left(\begin{aligned} &\frac{\partial^k}{\partial X_1^k} \frac{\partial f}{\partial u}(0) + R^k \Big(\frac{\partial^j}{\partial X_1^j} \frac{\partial f}{\partial u}(0), \\ &\qquad \frac{\partial^j}{\partial X_1^j} \frac{\partial f}{\partial X_i}(0); j < k, 1 \leq i \leq 2n \Big) \end{aligned} \right)_{2 \leq k \leq m-1}$$

to a m-jet $j_0^m f$.

This linear mapping being obviously surjective, the conclusion follows.

□

Theorem 4 follows from Lemma 1 combined with the classical Thom's transversality Theorem. □

Remark 1. In the computations in the jet space, only $f(X, 0)$, $\frac{\partial f}{\partial u}(X, 0)$, and their derivatives with respect to X appear. Thus the statement of Theorem 4 still holds if we replace $C^\infty(\mathbb{R}^{3n}, \mathbb{R})$ by the set of polynomial functions of u with coefficients in $C^\infty(\mathbb{R}^{2n}, \mathbb{R})$, or, even better, by the space of functions $f(X, u)$ differentiable with respect to u at $u = 0$ (and such that $f(X, 0)$ and $\frac{\partial f}{\partial u}(X, 0)$ are smooth). On the other hand, since the set O is open, it is also possible to replace $C^\infty(\mathbb{R}^{3n}, \mathbb{R})$ by any of its open subsets, for instance by the set of strictly convex functions w.r.t. u in $C^\infty(\mathbb{R}^{3n}, \mathbb{R})$.

Proof of Theorem 5

We consider a control system where the control acts linearly on the acceleration, with as many inputs as degrees of freedom:

$$\ddot{x} = \phi(x, \dot{x}) + N(x)u,$$

where

- x belongs to $\mathbb{R}^n$ (or to a n-dimensional differentiable manifold);
- the control $u \in \mathbb{R}^n$ is bounded: $u_i^- \leq u_i \leq u_i^+$ with $u_i^- < 0$, $u_i^+ > 0$;
- $\phi \in C^\infty(\mathbb{R}^{2n}, \mathbb{R}^n)$;
- for every x the $(n \times n)$ matrix $N(x)$ is invertible and $x \mapsto N(x)$ is C^∞.

Setting $X = (x, y)$, we rewrite the system as:

$$\dot{X} = F(X) + \sum_{i=1}^{n} u_i b_i(X), \; X \in \mathbb{R}^{2n}, \; u \in U \subset \mathbb{R}^n, \tag{4}$$

where F and $b_1, \ldots, b_n$ are vector fields on $\mathbb{R}^{2n}$.

An equilibrium of this system is a stationary trajectory $X \equiv X^0$, associated to a control $u \equiv u^0$ with:

$$F(X^0) + \sum_i u_i^0 b_i(X^0) = 0.$$

Fix a "source-point" $X^0 \in \mathbb{R}^{2n}$, a "target-point" $X^1 \in \mathbb{R}^{2n}$, and a time $T > 0$. Given a function f on $\mathbb{R}^{3n}$, we define the following optimal control problem:

$(\mathcal{P}_f)$ minimize the cost $J(u) = \int_0^T f(X, u)dt$
among the trajectories of Equation 4 joining X^0 to X^1.

We will restrict to functions $f(X, u)$ in $\mathcal{SC}$, the set of C^∞ functions from $\mathbb{R}^{2n} \times \mathbb{R}^n$ to $\mathbb{R}$ which are strictly convex with respect to u (in the strong sense, of course, that the Hessian is positive definite). The precise result we show is more than Theorem 5: it shows that the bad subset is very small (has infinite codimension).

Theorem 1. *There exists an open and dense subset O' of $\mathcal{SC}$ (endowed with the C^∞ Whitney topology) such that, if $f \in O'$, then $(\mathcal{P}_f)$ does not admit minimizing controls u with a component u_i vanishing on a subinterval of* $[0, T]$, *except maybe if the associated trajectory on the subinterval is an equilibrium of the system. In addition, for every integer N, the set O' can be chosen so that its complement has codimension greater than N.*

Of course we assume $T > T_{\min}$, the minimum time. Again the proof is based upon Thom's transversality theorem, we will then make the computations in the spaces of jets. For a positive integer N and a pair $(X, u) \in \mathbb{R}^{2n} \times \mathbb{R}^n$, we denote by $\mathcal{J}^N_{(X,u)}$ the space of N-jets at (X, u) of functions in $C^\infty(\mathbb{R}^{3n}, \mathbb{R})$.

Lemma 2. *Let $f \in C^{\infty}_{sc}(\mathbb{R}^{3n}, \mathbb{R})$. Assume that the trajectory (X, u) minimizing $(\mathcal{P}_f)$ satisfies, on a subinterval I of $[0, T]$:*

- $u_{i_0} \equiv 0$ *for some* $i_0 \in \{1, \dots, n\}$*;*
- $\dot{X} \neq 0$ *(i.e., the restriction $X_{|I}$ contains no equilibrium of the system).*

Then there exists $t \in I$ such that the N-jet $j^N_{(X(t),u(t))} f$ belongs to a semi-algebraic subset of $\mathcal{J}^N_{(X(t),u(t))}$ of codimension greater than $N - 2n$.

Proof. Recall that, under the hypothesis of the lemma, there is a trajectory (X, u) minimizing $(\mathcal{P}_f)$. Moreover this trajectory is not the projection of a singular extremal, and its associated control u is continuous. Thus, applying Pontryagin's Maximum Principle on I, there exists a C^1 function $P = (p, q) : I \to \mathbb{R}^n \times \mathbb{R}^n$ such that, for all $t \in I$:

(P1) $\quad \dot{P}(t)^T = -\frac{\partial H}{\partial X}(X(t), P(t), u(t))$,

(P2) $\quad H(X(t), P(t), u(t)) = \max_{v \in U} H(X(t), P(t), v)$,

where H is the normal Hamiltonian of the problem,

$$H(X, P, \lambda, u) = p^T y + q^T(\phi(X) + N(x)u) - f(X, u).$$

From (P1), the following holds on the interval I:

$$\begin{cases} \dot{p}^T &= -q^T \frac{\partial \phi}{\partial x}(X) - \frac{\partial f}{\partial x}(X, u), \\ \dot{q}^T &= -p^T - q^T \frac{\partial \phi}{\partial y}(X) - \frac{\partial f}{\partial y}(X, u). \end{cases} \tag{5}$$

On the other hand, (P2) implies that, for every $t \in I$, $u(t)$ satisfies the Karush-Kuhn-Tucker conditions: there exist Lagrange multipliers $\lambda^+(t), \lambda^-(t)$ in $\mathbb{R}^n$ such that:

$$\begin{cases} N(x(t))^T q(t) - \dfrac{\partial f}{\partial u}(X(t), u(t))^T - \lambda^+(t) - \lambda^-(t) = 0, \\ \lambda_i^+(t), \lambda_i^-(t) \geq 0, \quad i = 1, \dots, n, \\ \lambda_i^+(t)(u_i(t) - u_i^+) = \lambda_i^-(t)(u_i(t) - u_i^-) = 0, \ i = 1, \dots, n. \end{cases}$$

Since the control u is continuous, we may assume without lack of generality that there exist a nonempty subinterval J of I and an integer $m \in \{0, \dots, n-1\}$ such that:

- for $i = 1, \dots, m$, we have $u_i(t) \in]u_i^-, u_i^+[$ for every $t \in J$; in this case $\lambda_i^+ \equiv \lambda_i^- \equiv 0$ and,
$$(N(x)^T q)_i = \frac{\partial f}{\partial u_i}(X, u) \quad \text{on } J;$$
- for $i = m + 1, \dots, n - 1$, u_i is constant on J and equals to u_i^- or u_i^+;
- $u_n \equiv 0$ vanishes on J (i.e., $i_0 = n$); as a consequence, $\lambda_n^+ = \lambda_n^- = 0$ and,
$$(N(x)^T q)_n = \frac{\partial f}{\partial u_n}(X, u) \quad \text{on } J.$$

Denote by $\bar{v} = (v_1, \ldots, v_m)$ the first m coordinates of a vector $v \in \mathbb{R}^n$. Then the minimizing control can be written as $u(t) = (\bar{u}(t), u^0)$, where $u^0 \in \mathbb{R}^{n-m}$ is constant, and,

$$\overline{N(x)^T q} = \frac{\partial f}{\partial \bar{u}}(X, u)^T \quad \text{on } J. \tag{6}$$

Case 1. The matrix $\frac{\partial^2 f}{\partial \bar{u}^2}(X, u)$ is invertible on a subinterval J' of J.

It results from the Implicit Functions Theorem applied to Equation 6 that $\bar{u}$ is C^1 on J' and, for all $t \in J'$,

$$\begin{aligned} \dot{\bar{u}}(t) &= \tfrac{\partial^2 f}{\partial \bar{u}^2}(X(t), u(t))^{-1} \Big(\tfrac{d}{dt} \overline{N(x(t))^T q(t)} \\ &\quad - (L_F - \textstyle\sum_i u_i(t) L_{b_i}) \tfrac{\partial f}{\partial \bar{u}}(X(t), u(t))^T \Big), \end{aligned}$$

where L_F and L_{b_i} denote the Lie derivative with respect to respectively F and b_i. We use Equation 5 to eliminate $\dot{q}(t)$ in the expression

$$\frac{d}{dt} \overline{N(x(t))^T q(t)} = \overline{DN(x(t))^T (y) q(t)} + \overline{N(x(t))^T \dot{q}(t)},$$

and we obtain:

$$\begin{aligned} \dot{\bar{u}}(t) &= Q_{X(t)} \Big(p(t), q(t), u(t); \\ &\qquad \tfrac{\partial^2 f}{\partial \bar{u}_i \partial \bar{u}_j}, \tfrac{\partial^2 f}{\partial \bar{u}_i \partial X_j}, \tfrac{\partial f}{\partial X_i} \text{ at } (X(t), u(t)) \Big), \end{aligned} \tag{7}$$

where Q_X is a rational function depending smoothly on X.

Fix now $s \in J'$. Since $\dot{X}(t) = F(X(t)) + \sum_i u_i(t) b_i(X(t))$ is never vanishing on J', we may assume, up to a local change of the coordinates $X = (X_1, \ldots, X_{2n})$ on $\mathbb{R}^{2n}$ near $X(s)$, that $F(X) + \sum_i u_i(s) b_i(X) = \frac{\partial}{\partial X_1}$. Differentiating $(N(x)^T q)_n = \frac{\partial f}{\partial u_n}(X, u)$ with respect to time near $t = s$ leads to

$$\begin{aligned} \tfrac{d}{dt}(N(x(t))^T q(t))_n &= \tfrac{\partial^2 f}{\partial u_n \partial X_1}(X(t), u(t)) \\ &\quad + \textstyle\sum_i \Delta u_i^s(t) L_{b_i} \tfrac{\partial f}{\partial u_n}(X(t), u(t)) \\ &\quad + \textstyle\sum_{i=1}^m \tfrac{\partial^2 f}{\partial u_n \partial \bar{u}_i}(X(t), u(t)) \dot{\bar{u}}_i(t), \end{aligned}$$

where $\Delta u^s(t) = u(t) - u(s)$. We substitute the expressions Equation 7 of $\dot{\bar{u}}(t)$ and Equation 5 of $\dot{q}_n$ into this equation, and we obtain, for t near s,

$$\begin{aligned} &\tfrac{\partial^2 f}{\partial u_n \partial X_1} + R_X^1 (\Delta u^s \tfrac{\partial^2 f}{\partial u_n \partial X_i}, \\ &\qquad \tfrac{\partial^2 f}{\partial u_i \partial u_j}, \tfrac{\partial^2 f}{\partial \bar{u}_i \partial X_j}, \tfrac{\partial f}{\partial \alpha_i}, p, q, u) = 0, \end{aligned}$$

where R_X^1 is a rational function with coefficients depending smoothly on X, and α_i, $1 \leq i \leq 3n$, denotes the ith component of the vector $\alpha = (X, u)$.

Successive derivations (with substitution of $\dot{\bar{u}}(t)$ by Equation 7 and of $\dot{p}$ and $\dot{q}$ by Equation 5 at each step) and evaluation of the derivatives at $t = s$ lead to a system of equations of the form, for $k \geq 1$,

$$\begin{aligned} &\tfrac{\partial^{k+1} f}{\partial u_n \partial X_1^k}(X(s), u(s)) + R^k(P(s), \\ &\qquad \tfrac{\partial^j f}{\partial \alpha_{i_1} \cdots \partial \alpha_{i_j}}(X(s), u(s)); j \leq k+1) = 0, \end{aligned}$$

where R^k is a rational function, and if $j = k+1$ then at least one of the α_{i_ℓ} is a $\bar{u}_i$.

Let Ω_1^N be the set of N-jets $j^N_{(X(s),u(s))} f$ such that $\det(\frac{\partial^2 f}{\partial \bar{u}^2}(X(s),u(s))) \neq 0$. It is an open subset of $\mathcal{J}^N_{(X(s),u(s))}$.

We have proved that $(j^N_{(X(s),u(s))} f, P(s))$ belongs to $\psi_1^{-1}(0)$, where $\psi_1 : \Omega_1^N \times \mathbb{R}^{2n} \to \mathbb{R}^{N-1}$ is the rational mapping which to a N-jet $j^N_{(X(s),u(s))} g \in \Omega_1^N$ and a vector $P \in \mathbb{R}^{2n}$ associates

$$\left(\begin{array}{c} \frac{\partial^{k+1} g}{\partial u_n \partial X_1^k}(X(s),u(s)) + R^k(P, \\ \qquad \frac{\partial^j g}{\partial \alpha_1 \cdots \partial \alpha_j}(X(s),u(s)); j \leq k+1) \end{array} \right)_{1 \leq k \leq N-1}.$$

This mapping is clearly surjective, therefore $\psi_1^{-1}(0)$ is a semi-algebraic subset of $\mathcal{J}^N_{(X(s),u(s))} \times \mathbb{R}^{2n}$ of codimension $N-1$. The projection of $\psi_1^{-1}(0)$ on $\mathcal{J}^N_{(X(s),u(s))}$ is then a semi-algebraic subset of codimension greater than $N-2n$, which moreover contains the N-jet $j^N_{(X(s),u(s))} f$.

Case 2. The matrix $\frac{\partial^2 f}{\partial \bar{u}^2}(X,u)$ is never invertible on J.

In order to show that $\bar{u}$ is C^1 and to derive an expression for $\dot{\bar{u}}$, we need to introduce some notations. We define inductively a sequence of mappings $V^\ell : \mathbb{R}^{2n} \times \mathbb{R}^n \to \mathbb{R}^m$ by:

- $V^0 = \dfrac{\partial f}{\partial \bar{u}}^T$
- for a positive integer ℓ, the components of V^ℓ are:

$$V_k^\ell = \begin{cases} V_k^{\ell-1} & \text{if } 1 \leq k \leq r_\ell, \\ \det \left(\dfrac{\partial V_i^{\ell-1}}{\partial \bar{u}_j} \right)_{i,j=1,\ldots,r_\ell,k} & \text{if } r_\ell + 1 \leq k \leq m, \end{cases}$$

  where $r_\ell = r_\ell(X,u)$ is the rank of the matrix $\frac{\partial V^{\ell-1}}{\partial \bar{u}}(X,u)$.

By hypothesis, $r_1(X(t),u(t))$ is smaller than m for $t \in J$. Since $X(\cdot)$ and $u(\cdot)$ are continuous, up to a permutation of the indices $\{1,\ldots,m\}$, there is a subinterval J' of J such that, for any $\ell \geq 1$,

- the rank $r_\ell(X(t),u(t))$ is constant on J';
- the function

$$\delta_\ell(X(t),u(t)) = \det \left(\frac{\partial V_i^{\ell-1}}{\partial \bar{u}_j}(X(t),u(t)) \right)_{1 \leq i,j \leq r_\ell}$$

  is never vanishing on J';

- if $r_\ell < m$, then

$$V^\ell(X(t),u(t)) \quad = ((N(x(t))^T q(t))_1, \dots, (N(x(t))^T q(t))_{r_1}, 0, \dots, 0) \text{ for all } t \in J'.$$

Notice that an easy induction shows the following expression:

$$V^\ell_k = \delta_1 \dots \delta_\ell \frac{\partial^{\ell+1} f}{\partial \bar{u}_k^{\ell+1}} + G^{k,\ell}, \tag{8}$$

where $G^{k,\ell}$ is a polynomial function of the derivatives of the form $\frac{\partial^j f}{\partial \bar{u}_{i_1} \cdots \partial \bar{u}_{i_j}}$, with $j \leq \ell+1$, each $i_l \leq k$, and $\sum_l i_l < k(\ell+1)$.

Denote by L the largest integer such that $r_L < m$ (we set $L = +\infty$ if the latter condition is always satisfied). Then, for $\ell = 1, \dots, L$, $V^\ell_m(X,u) \equiv 0$ on J'. If moreover $L < \infty$, there holds on J',

$$V^L(X,u) \quad = ((N(x)^T q)_1, \dots, (N(x)^T q)_{r_1}, 0, \dots, 0) \quad \text{and} \quad \frac{\partial V^L}{\partial \bar{u}}(X,u) \text{ invertible,}$$

with $u(\cdot) = (\bar{u}(\cdot), u^0)$. It then results from the Implicit Functions Theorem that $\bar{u}$ is C^1 on J'. Following exactly the argument of Case 1, we obtain a system of equations of the form, for a fixed $s \in J'$,

$$\frac{\partial^{k+1} f}{\partial u_n \partial X_1^k}(X(s),u(s)) + R'_k = 0, \quad k \geq 1,$$

where R'_k is a rational function of $P(s)$ and of derivatives $\frac{\partial^j f}{\partial \alpha_{i_1} \cdots \partial \alpha_{i_j}}(X(s),u(s))$ such that $j \leq k+L$ and, if one of the α_{i_ℓ} is u_n, then $j \leq k+1$ and $j = k+1$ implies that at least one of the other $\alpha_{i_{\ell'}}$ is a $\bar{u}_i$.

Set $M = \min(L, N-1)$. Let Ω_2^N be the set of N-jets $j^N_{(X(s),u(s))}f$ such that:

$$\delta_1(X(s),u(s)) \dots \delta_M(X(s),u(s)) \neq 0.$$

It is thus an open subset of $\mathcal{J}^N_{(X(s),u(s))}$.

We have proved that $(j^N_{(X(s),u(s))}f, P(s))$ belongs to $\psi_2^{-1}(0)$, where $\psi_2 : \Omega_2^N \times \mathbb{R}^{2n} \to \mathbb{R}^{N-1}$ is the rational mapping which to $(j^N_{(X(s),u(s))}f, P(s))$ associates

$$\left(\left(\delta_1 \dots \delta_\ell \frac{\partial^{\ell+1} f}{\partial \bar{u}_k^{\ell+1}}(X(s),u(s)) + G^{k,\ell} \right)_{1 \leq \ell \leq M}, \left(\frac{\partial^{k+1} f}{\partial u_n \partial X_1^k}(X(s),u(s)) + R'_k \right)_{1 \leq k \leq N-M-1} \right).$$

This mapping is clearly surjective, therefore $\psi_2^{-1}(0)$ is a semi-algebraic subset of $\mathcal{J}^N_{(X(s),u(s))} \times \mathbb{R}^{2n}$ of codimension $N-1$. The projection of $\psi_2^{-1}(0)$ on $\mathcal{J}^N_{(X(s),u(s))}$ is then a semi-algebraic subset of codimension greater than $N-2n$, which contains the N-jet $j^N_{(X(s),u(s))}f$.

□

Theorem 1 follows from Lemma 2 combined with standard transversality arguments.

Computation of Extremals in the 2-dof Case

We use the stratification of the (u_1, u_2)-plane with respect to the "sign of coordinates". Thus we have the following analysis.

1. In the strata $u_1, u_2 > 0$, the maximum of $\bar{\mathcal{H}}(u_1, u_2)$ is solution of the following system (setting $s_1 = -1$, $s_2 = -1$) :

$$
\begin{aligned}
&0 = \frac{\partial \bar{\mathcal{H}}}{\partial u_1} = \\
&s_1.|y_1| - 2\alpha_1 \bar{H}_{11}(\bar{H}_{11}.[u_1 - G_1 + h.(y_2^2 + 2y_1y_2) \\
&- B_{11}y_1 - B_{12}y_2] \\
&+ \bar{H}_{12}.[u_2 - G_2 - h.y_1^2 - B_{21}y_1 - B_{22}y_2]) \\
&- 2\alpha_2 \bar{H}_{21}(\bar{H}_{21}.[u_1 - G_1 + h.(y_2^2 + 2y_1y_2) \\
&- B_{11}y_1 - B_{12}y_2] \\
&+ \bar{H}_{22}.[u_2 - G_2 - h.y_1^2 - B_{21}y_1 - B_{22}y_2]) \\
&+ q_1\bar{H}_{11} + q_2\bar{H}_{21}
\end{aligned}
$$

and,

$$
\begin{aligned}
&0 = \frac{\partial \bar{\mathcal{H}}}{\partial u_2} = \\
&s_2.|y_2| - 2\alpha_1 \bar{H}_{12}(\bar{H}_{11}.[u_1 - G_1 + h.(y_2^2 + 2y_1y_2) \\
&- B_{11}y_1 - B_{12}y_2] \\
&+ \bar{H}_{12}.[u_2 - G_2 - h.y_1^2 - B_{21}y_1 - B_{22}y_2]) \\
&- 2\alpha_2 \bar{H}_{22}(\bar{H}_{21}.[u_1 - G_1 + h.(y_2^2 + 2y_1y_2) \\
&- B_{11}y_1 - B_{12}y_2] \\
&+ \bar{H}_{22}.[u_2 - G_2 - h.y_1^2 - B_{21}y_1 - B_{22}y_2]) \\
&+ q_1\bar{H}_{12} + q_2\bar{H}_{22}.
\end{aligned}
$$

Regrouping the $u_i's$ all together, we get:

$$
\begin{aligned}
&(2\alpha_1\bar{H}_{11}^2 + 2\alpha_2\bar{H}_{21}^2)u_1 + (2\alpha_1\bar{H}_{11}\bar{H}_{12} + 2\alpha_2\bar{H}_{21}\bar{H}_{22})u_2 \\
&= s_1.|y_1| - 2\alpha_1\bar{H}_{11}(\bar{H}_{11}.[-G_1 + h.(y_2^2 + 2y_1y_2) \\
&- B_{11}y_1 - B_{12}y_2] \\
&+ \bar{H}_{12}.[-G_2 - h.y_1^2 - B_{21}y_1 - B_{22}y_2]) \\
&- 2\alpha_2\bar{H}_{21}(\bar{H}_{21}.[-G_1 + h.(y_2^2 + 2y_1y_2) \\
&- B_{11}y_1 - B_{12}y_2] \\
&+ \bar{H}_{22}.[-G_2 - h.y_1^2 - B_{21}y_1 - B_{22}y_2]) \\
&+ q_1\bar{H}_{11} + q_2\bar{H}_{21}
\end{aligned}
$$

and,

$$
\begin{aligned}
&(2\alpha_1\bar{H}_{12}\bar{H}_{11} + 2\alpha_2\bar{H}_{22}\bar{H}_{21})u_1 + (2\alpha_1\bar{H}_{12}^2 + 2\alpha_2\bar{H}_{22}^2)u_2 \\
&= s_2.|y_2| - 2\alpha_1\bar{H}_{12}(\bar{H}_{11}.[-G_1 + h.(y_2^2 + 2y_1y_2) \\
&- B_{11}y_1 - B_{12}y_2] \\
&+ \bar{H}_{12}.[-G_2 - h.y_1^2 - B_{21}y_1 - B_{22}y_2]) \\
&- 2\alpha_2\bar{H}_{22}(\bar{H}_{21}.[-G_1 + h.(y_2^2 + 2y_1y_2) \\
&- B_{11}y_1 - B_{12}y_2] \\
&+ \bar{H}_{22}.[-G_2 - h.y_1^2 - B_{21}y_1 - B_{22}y_2]) \\
&+ q_1\bar{H}_{12} + q_2\bar{H}_{22},
\end{aligned}
$$

Which is a system of the general form:

$$
\begin{aligned}
s_1.|y_1| + C_1 &= (2\alpha_1\bar{H}_{11}^2 + 2\alpha_2\bar{H}_{21}^2)u_1 \\
&\quad +(2\alpha_1\bar{H}_{11}\bar{H}_{12} + 2\alpha_2\bar{H}_{21}\bar{H}_{22})u_2 \\
s_2.|y_2| + C_2 &= (2\alpha_1\bar{H}_{12}\bar{H}_{11} + 2\alpha_2\bar{H}_{22}\bar{H}_{21})u_1 \\
&\quad +(2\alpha_1\bar{H}_{12}^2 + 2\alpha_2\bar{H}_{22}^2)u_2.
\end{aligned}
$$

The solutions follow:

$$
\begin{aligned}
u_1 &= \frac{(\alpha_1\bar{H}_{12}^2 + \alpha_2\bar{H}_{22}^2)(C_1 + s_1.|y_1|)}{2\alpha_1\alpha_2(\bar{H}_{11}\bar{H}_{22} - \bar{H}_{12}\bar{H}_{21})^2} \\
&\quad - \frac{(\alpha_2\bar{H}_{21}\bar{H}_{22} + \alpha_1\bar{H}_{12}\bar{H}_{11})(C_2 + s_2.|y_2|)}{2\alpha_1\alpha_2(\bar{H}_{11}\bar{H}_{22} - \bar{H}_{12}\bar{H}_{21})^2} \\
u_2 &= \frac{-(\alpha_1\bar{H}_{11}\bar{H}_{12} + \alpha_2\bar{H}_{21}\bar{H}_{22})(C_1 + s_1.|y_1|)}{2\alpha_1\alpha_2(\bar{H}_{11}\bar{H}_{22} - \bar{H}_{12}\bar{H}_{21})^2} \\
&\quad + \frac{(\alpha_1\bar{H}_{11}^2 + \alpha_2\bar{H}_{21}^2)(C_2 + s_2.|y_2|)}{2\alpha_1\alpha_2(\bar{H}_{11}\bar{H}_{22} - \bar{H}_{12}\bar{H}_{21})^2}
\end{aligned}
\tag{9}
$$

2. In the strata $u_1 > 0$ and $u_2 < 0$, the maximum is solution of the same system, and has the same expression (Equation 9), but taking $s_1 = -1$ and $s_2 = +1$.

3-4. In the stratas S_3, S_4, corresponding respectively to ($u_1 < 0$, $u_2 < 0$), ($u_1 < 0$, $u_2 > 0$), we get the same expression taking respectively ($s_1 = +1$, $s_2 = -1$), ($s_1 = +1$, $s_2 = +1$).

5. For the strata $u_1 = 0$ and $u_2 > 0$, we set $s_2 = -1$. The maximum is given by:

$$
\begin{aligned}
&0 = \frac{\partial\bar{\mathcal{H}}}{\partial u_2} = \\
&s_2.|y_2| - 2\alpha_1\bar{H}_{12}(\bar{H}_{11}.[-G_1 + h.(y_2^2 + 2y_1y_2) \\
&- B_{11}y_1 - B_{12}y_2] \\
&+ \bar{H}_{12}.[u_2 - G_2 - h.y_1^2 - B_{21}y_1 - B_{22}y_2]) \\
&- 2\alpha_2\bar{H}_{22}(\bar{H}_{21}.[-G_1 + h.(y_2^2 + 2y_1y_2) \\
&- B_{11}y_1 - B_{12}y_2] \\
&+ \bar{H}_{22}.[u_2 - G_2 - h.y_1^2 - B_{21}y_1 - B_{22}y_2]) \\
&+ q_1\bar{H}_{12} + q_2\bar{H}_{22}.
\end{aligned}
$$

Regrouping the terms in u_2:

$$\begin{aligned}
&(2\alpha_1\bar{H}_{12}^2 + 2\alpha_2\bar{H}_{22}^2)u_2 \\
&= s_2.|y_2| - 2\alpha_1\bar{H}_{12}(\bar{H}_{11}.[-G_1 + h.(y_2^2 + 2y_1y_2) \\
&- B_{11}y_1 - B_{12}y_2] \\
&+ \bar{H}_{12}.[-G_2 - h.y_1^2 - B_{21}y_1 - B_{22}y_2]) \\
&- 2\alpha_2\bar{H}_{22}(\bar{H}_{21}.[-G_1 + h.(y_2^2 + 2y_1y_2) \\
&- B_{11}y_1 - B_{12}y_2] \\
&+ \bar{H}_{22}.[-G_2 - h.y_1^2 - B_{21}y_1 - B_{22}y_2]) \\
&+ q_1\bar{H}_{12} + q_2\bar{H}_{22},
\end{aligned}$$

or,

$$(2\alpha_1\bar{H}_{12}^2 + 2\alpha_2\bar{H}_{22}^2)u_2 = s_2.|y_2| + C_2.$$

Therefore:

$$u_2 = \frac{s_2.|y_2| + C_2}{2\alpha_1\bar{H}_{12}^2 + 2\alpha_2\bar{H}_{22}^2}.$$

6. In the strata $u_1 = 0$ and $u_2 < 0$ the expression is similar, with $s_2 = +1$.

7. In the strata $u_1 > 0$ and $u_2 = 0$, we set $s_1 = -1$.

$$\begin{aligned}
&0 = \frac{\partial\bar{\mathcal{H}}}{\partial u_1} = \\
&s_1.|y_1| - 2\alpha_1\bar{H}_{11}(\bar{H}_{11}.[u_1 - G_1 + h.(y_2^2 + 2y_1y_2) \\
&- B_{11}y_1 - B_{12}y_2] \\
&+ \bar{H}_{12}.[-G_2 - h.y_1^2 - B_{21}y_1 - B_{22}y_2]) \\
&- 2\alpha_2\bar{H}_{21}(\bar{H}_{21}.[u_1 - G_1 + h.(y_2^2 + 2y_1y_2) \\
&- B_{11}y_1 - B_{12}y_2] \\
&+ \bar{H}_{22}.[-G_2 - h.y_1^2 - B_{21}y_1 - B_{22}y_2]) \\
&+ q_1\bar{H}_{11} + q_2\bar{H}_{21}.
\end{aligned}$$

Regrouping the u_1 terms:

$$\begin{aligned}
&(2\alpha_1\bar{H}_{11}^2 + 2\alpha_2\bar{H}_{21}^2)u_1 \\
&= s_1.|y_1| - 2\alpha_1\bar{H}_{11}(\bar{H}_{11}.[-G_1 + h.(y_2^2 + 2y_1y_2) \\
&- B_{11}y_1 - B_{12}y_2] \\
&+ \bar{H}_{12}.[-G_2 - h.y_1^2 - B_{21}y_1 - B_{22}y_2]) \\
&- 2\alpha_2\bar{H}_{21}(\bar{H}_{21}.[-G_1 + h.(y_2^2 + 2y_1y_2) \\
&- B_{11}y_1 - B_{12}y_2] \\
&+ \bar{H}_{22}.[-G_2 - h.y_1^2 - B_{21}y_1 - B_{22}y_2]) \\
&+ q_1\bar{H}_{11} + q_2\bar{H}_{21},
\end{aligned}$$

or,

$$(2\alpha_1\bar{H}_{11}^2 + 2\alpha_2\bar{H}_{21}^2)u_1 = s_1.|y_1| + C_1.$$

From what:

$$u_1 = \frac{s_1.|y_1| + C_1}{2\alpha_1\bar{H}_{11}^2 + 2\alpha_2\bar{H}_{21}^2}.$$

8. In the strata $u_1 < 0$ and $u_2 = 0$, we get the same expression with $s_1 = +1$.
9. On the last strata $u_1 = u_2 = 0$, the maximum is obviously $u_1 = u_2 = 0$.

Notice also that we know (Theorem 3) that the optimal control is continuous.

Then, we integrate Pontryagin's equations by finding the maximum of the Hamiltonian within the 9 expressions above, and checking in which region it is.

A trial and error procedure on the initial adjoint vector does the job.
